# Highly Pathogenic Avian Influenza (HPAI) H5 Clade 2.3.4.4b Virus Infection in Birds and Mammals

**DOI:** 10.3390/ani14091372

**Published:** 2024-05-02

**Authors:** Giulia Graziosi, Caterina Lupini, Elena Catelli, Silvia Carnaccini

**Affiliations:** 1 Department of Veterinary Medical Sciences, University of Bologna, Ozzano dell’Emilia, 40064 Bologna, Italy; giulia.graziosi2@unibo.it (G.G.); caterina.lupini@unibo.it (C.L.); elena.catelli@unibo.it (E.C.); 2 Department of Population Health, College of Veterinary Medicine, University of Georgia, Athens, GA 30602, USA

**Keywords:** avian influenza, pathobiology, wild birds, poultry, wild mammals, domestic pets, virus spillover, public health

## Abstract

**Simple Summary:**

Avian influenza viruses are highly contagious respiratory viruses that severely impact bird populations, causing significant morbidity, mortality, and economic losses in the poultry industry worldwide. Particularly concerning are the Asian-origin H5 subtype highly pathogenic avian influenza viruses of clade 2.3.4.4b, which emerged in 2013 and have since spread across Asia, Europe, Africa, and America, leading to outbreaks in various poultry and animal species. The unique epidemiological and pathobiological characteristics specific to clade 2.3.4.4b viruses are discussed, emphasizing their distinct nature compared to other clades. Wild waterfowl, acting as reservoirs, frequently carry these viruses, posing threats not only to their populations but also to other wild bird species, including endangered ones. Furthermore, an increasing number of clade 2.3.4.4b virus infections in wild or domestic mammalian species raises significant concerns about potential spillover events to humans. This review highlights the diverse outcomes of HPAI infections in different hosts, ranging from asymptomatic cases to fatal infection, influenced by host and virus-related factors. Understanding these complexities is vital for developing effective strategies to mitigate the impact of AIVs, safeguard poultry production, protect wildlife, and prevent potential public health crises.

**Abstract:**

Avian influenza viruses (AIVs) are highly contagious respiratory viruses of birds, leading to significant morbidity and mortality globally and causing substantial economic losses to the poultry industry and agriculture. Since their first isolation in 2013–2014, the Asian-origin H5 highly pathogenic avian influenza viruses (HPAI) of clade 2.3.4.4b have undergone unprecedented evolution and reassortment of internal gene segments. In just a few years, it supplanted other AIV clades, and now it is widespread in the wild migratory waterfowl, spreading to Asia, Europe, Africa, and the Americas. Wild waterfowl, the natural reservoir of LPAIVs and generally more resistant to the disease, also manifested high morbidity and mortality with HPAIV clade 2.3.4.4b. This clade also caused overt clinical signs and mass mortality in a variety of avian and mammalian species never reported before, such as raptors, seabirds, sealions, foxes, and others. Most notably, the recent outbreaks in dairy cattle were associated with the emergence of a few critical mutations related to mammalian adaptation, raising concerns about the possibility of jumping species and acquisition of sustained human-to-human transmission. The main clinical signs and anatomopathological findings associated with clade 2.3.4.4b virus infection in birds and non-human mammals are hereby summarized.

## 1. Introduction

**Figure 1:**
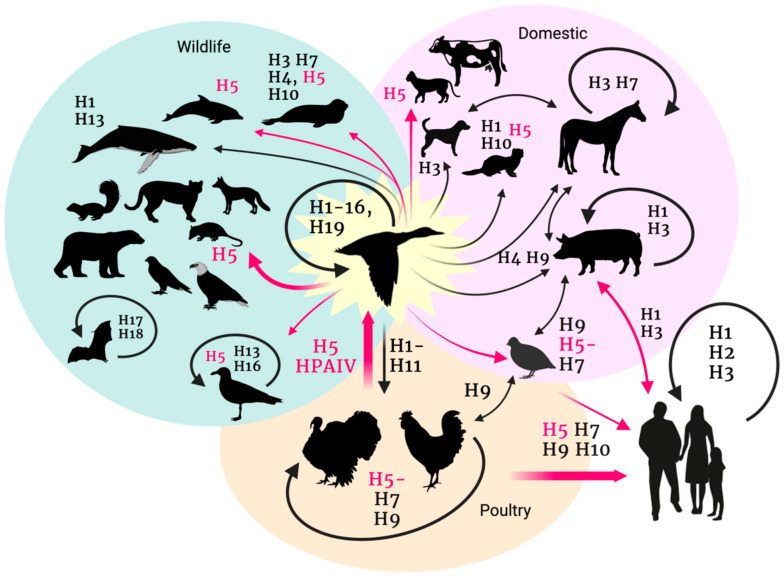
Avian influenza transmission flow from the natural reservoir (aquatic birds) to poultry, humans, and other animal species. Figure generated with BioRender.com, accessed on 28 April 2024.

Avian influenza viruses (AIVs) belong to the species *Alphainfluenzavirus influenzae* (previously influenza A virus or FLUAV), genus *Alphainfluenzavirus*, family Orthomyxoviridae [1,2]. AIVs are highly contagious respiratory viruses of birds, responsible for high morbidity and mortality depending on the strain considered. These are single-stranded, negative-sense, segmented, and enveloped RNA viruses, which include eight gene segments coding for at least 11 structural and non-structural/regulatory proteins [3,4,5]. The polymerase complex, composed of the polymerase basic protein 1 (PB1), polymerase basic protein 2 (PB2), and the polymerase acidic protein (PA), altogether with the nucleoprotein (NP), the matrix protein 1 (M1), the non-structural protein 1 (NS1), and the nuclear export protein (NEP) are found inside the lipid envelope, while the membrane ion channel (M2), the hemagglutinin (HA), and the neuraminidase (NA) are embedded within the envelope. The molecular properties of HA and NA proteins determine FLUAV classification into subtypes [6]. A total of 19 HA (H1–H19) and 11 NA (N1–N11) have been recognized, with H17N10 and H18N11 “flu-like” subtypes exclusively isolated in bats (Figure 1) [7,8,9,10,11]. 

Wild waterfowl (Anseriformes, Charadriiformes) are considered the natural reservoir of 17 of 19 HA (H1-16 and H19) and 9 of 11 NA (N1-9) AIV subtypes, and they carry AIVs mostly subclinically [12]. Spillover events of AIVs from wild birds to domestic poultry typically result in asymptomatic or mild to moderate respiratory disease, diarrhea, and drop in egg production. On occasion, the circulation of low pathogenic avian influenza (LPAI) H5 and H7 virus subtypes in poultry leads to virus evolution and increased disease severity with up to 100% mortality rates. Therefore, based on the disease severity in chickens, AIVs are further classified as either low or highly pathogenic (LPAI or HPAI). H5 and H7 subtypes are the only two subtypes recognized as HPAIVs, which are reportable diseases to the World Organization for Animal Health (WOAH) [13,14,15]. Eurasian H5 HPAIVs are a serious concern for the WOAH since these viruses are zoonotic, potentially pandemic, and manifest increased host range and tissue tropism. 

Eurasian H5s are the immediate descendants of the A/goose/Guangdong/1/1996(Gs/Gd)-like H5N1 HPAI virus that was first detected in sick farmed geese in Guangdong province, China, in 1996 [16,17]. After becoming endemic in domestic poultry in Asia, HPAI viruses from this phylogenetic lineage have found a way back into the natural host (migratory aquatic birds), resulting in significant geographic spread worldwide to Asia, Africa, Europe, and America and causing significant losses to the poultry industry [18,19]. Since their first emergence, the HA gene of H5 Gs/Gd HPAIV has genetically diversified into an overabundance of clades and subclades. Among these, clade 2.3.4.4b has undergone explosive expansion in wild birds and domestic poultry and almost entirely replaced other circulating clades in just a short period of time [19,20,21]. Strains from clade 2.3.4.4b have been reported to cause severe systemic infections and high mortality rates among waterfowl, as well as mass mortality events in sea birds, marine mammals, and others [22]. As of March 2024, livestock and dairy herds have tested positive in Minnesota, Texas, Kansas, Michigan, and other States [23]. While sustained mammalian transmission has not been documented yet, the persistent recirculation of this clade in wild and domestic mammalian populations is concerning for potential virus adaptation and species jumps.

Barriers to interspecies adaptation include changes within the viral polymerase complex to achieve successful replication within mammalian cells and improved receptor binding activities of the HA and NA proteins [24]. The conformation of the HA protein is of particular importance, as it mediates binding to the host cell receptor through a receptor binding site (RBS) and the initiation of the virus cycle [25]. AIVs typically prefer α2,3-linked sialylated glycans present more abundantly in respiratory and gastrointestinal tracts of avian species, while mammalian FLUAVs preferably bind to α2,6-linked sialylated glycans predominant in mammalian tissues [26]. Upon endocytic uptake, the acidification of the endosome triggers a conformational change in the HA that mediates fusion between the viral envelope and the endosomal membrane. This allows the virus genome to be released into the cytoplasm [27]. Since HA is synthesized as an inactive precursor (HA0), this must be cleaved at a specific site, the cleavage site (CS), into HA1 and HA2 to become functional and induce membrane fusion and release of virus genome into the cytoplasm [27,28,29]. The amino acid motifs at the CS determine the spectrum of enzymes that can cleave and activate the HA [30]. Most LPAIVs contain a single arginine residue (monobasic) at the HA CS, which is cleaved extracellularly exclusively by trypsin or trypsin-like proteases, only secreted in lung epithelium [31] and intestinal tract [32], therefore mediating localized infections and hence milder disease [33]. However, when the CS contains multiple basic amino acids (polybasic CS), it can be cleaved by a family of serine proteases [34], which are ubiquitously expressed and allow virus replication in different cell types, causing an often fatal systemic disease [35,36]. Therefore, the CS sequence has been used by the WHO as a reference for classifying AIVs into pathotypes [37]. 

The HA protein also contains the major antigenic determinants targeted by the humoral host’s immune response, which undergoes heavy selection pressure [38,39,40,41,42,43]. When non-synonymous nucleotide substitutions accumulate in a process called *antigenic drift*, the resulting virus can escape neutralization by host antibodies [44]. More pronounced genetic variations can also result in changes in viral properties, such as receptor binding, replication, and transmission [45]. Furthermore, AIV’s segmented genome enables *antigenic shift*, a phenomenon that occurs between two or more viruses that infect the same cell and consists of the reassortment of the gene segments and the emergence of an entirely novel virus. In birds, reassortment events between different AIVs have contributed to the emergence of several novel emerging zoonotic subtypes with internal gene cassettes from other circulating subtypes [46]. This has also paved the way for the emergence of pandemic influenza in humans, including H1N1 (1918), H2N2 (1957), and H3N2 (1968) virus strains [47,48]. Therefore, given the significant implications for veterinary and public health, recognizing the early signs and clinicopathological manifestations of HPAI H5 Gs/Gd 2.3.4.4b clade viruses is crucial to promptly act with interventions to mitigate its spread across susceptible species. The review hereby presented aims to summarize the main clinical, pathological, and histopathological findings of clade 2.3.4.4b virus infections in main avian and mammalian hosts. 

## 2. H5 Genetic Evolution and Epidemiology

Due to their persistent circulation in poultry in Asia, Gs/Gd H5Nx HPAI viruses have found their way back to their natural reservoir, the wild aquatic bird species. Through waterfowl migrations, four waves of intercontinental transmission of Gs/Gd lineage H5Nx virus from Asia to other continents have been identified from 2003 onward [21]. This has led these viruses to undergo reassortment of the internal gene segments with other influenza viruses and genetic evolution of the hemagglutinin H5 gene. The genetic diversity of H5 Gs/Gd hemagglutinin has resulted in the identification of ten distinct clades (0 to 9) and multiple subclades, as outlined by a unified nomenclature [49,50]. Viruses from different clades have exhibited long-distance dissemination, and HPAI H5Nx viruses within the 2.3.4.4 clade have recently gathered significant attention due to their unprecedented global spread and their ability to infect a wide range of species, including not only domestic and wild birds, but also humans and other mammals [19,51]. Initially isolated from a Chinese live poultry market in 2010 [52], a subtype H5N8 virus of clade 2.3.4.4 caused multiple outbreaks in poultry and wild birds in South Korea [53]. Since its emergence, the HA gene of clade 2.3.4.4 has then undergone reassortment events with other avian influenza viruses from different regions, acquiring various NA subtypes, including N1–N6 and N8, as well as evolving and being reclassified in different phylogenetic clades and groups [54]. The first intercontinental spread of the H5 clade 2.3.4.4c (previously classified as 2.3.4.4a) to North America dates to 2014–2015, with H5N8 viruses introduced most likely via migratory birds through the Bering Strait to breeding grounds in Alaska [55]. After reassorting with LPAI gene segments from North American wild birds [56], an H5N2 reassortant virus of clade 2.3.4.4c became predominant and affected over 50 million birds in the U.S. in spring 2015 [19,21,57]. After sporadic detections in wild birds, the H5 clade 2.3.4.4c viruses in North America were last isolated in late 2016 [58]. During 2014/2015, HPAIVs clade 2.3.4.4 were simultaneously introduced into Europe and East Asia and have been circulating altogether with other clades, notably 2.3.2.1c in Cambodia and Vietnam, following fall bird migration [19,21,55,59]. 

Since their first detection in domestic ducks in Eastern China (2013) and South Korea (2014) [53,60], the H5Nx clade 2.3.4.4b viruses were isolated from wild birds in Qinghai Lake, China, in 2016 and spread on a large scale to Europe, Africa, Middle East, and Asia via migratory birds [19,61,62]. In Europe, 1207 HPAI H5Nx poultry outbreaks and 1590 wild bird mortality events were reported during the 2016/2017 virus epidemic, mostly due to H5N8 of clade 2.3.4.4b [63,64]. Sporadic outbreaks of HPAI H5Nx viruses were reported in Eastern Europe from 2018 to early 2020 until the emergence of a novel H5N1 strain of clade 2.3.4.4b in October 2020 in The Netherlands that will replace other H5 subtypes globally [65,66,67,68]. Since fall 2020, a surge in HPAI H5Nx cases due to 2.3.4.4b viruses has been observed across the Eurasian continent, with an unprecedented number of H5N1 outbreaks in poultry and cases in wild birds that peaked during the 2021–2022 epidemic season [69]. This led to the largest HPAI epidemic ever, with more than 2000 outbreaks in 37 European countries and over 40 million birds culled during 2021–2022 [70]. At least 19 different H5N1 clade 2.3.4.4b genotypes were characterized in Europe, originating from multiple inter- and intra-subtype reassortment events [70,71].

The transatlantic spread to North America of H5N1 clade 2.3.4.4b HPAI viruses occurred in late 2021. Notably, a die-off of domestic birds on the Atlantic coast of Canada was attributed to an HPAI H5N1 virus, which was also isolated from a great black-backed gull (*Larus marinus*). Strain characterization confirmed its placement within the 2.3.4.4b phylogenetic clade, signifying a new virus introduction into North America via migratory wild birds from Eurasia [72,73], possibly via Iceland [74]. A further intercontinental virus spread to North America then occurred via the Pacific and the North Atlantic-linked migratory flyways later in 2022 [75,76,77]. After the H5N1 virus spread westward and southward, extensive genetic reassortment with North American LPAIVs has brought to 10 different gene constellations composed of 6 major and 11 minor genotypes until April 2022. As of August 2023, the circulation of HPAI H5Nx viruses of clade 2.3.4.4b in the U.S. has resulted in 58 million domestic birds affected [78] and the detection of the virus in 7152 wild birds [79]. The H5 HPAIVs of clade 2.3.4.4b introduction to Central and South America occurred in late 2022, through migratory birds [80]. Die-offs of wild aquatic birds and wild mammals, and cases in poultry have therefore been reported in countries along the Pacific Americas flyway, such as Peru and Chile [81,82,83,84]. H5 HPAI cases have also been reported in countries on the east side of the Andes Mountains, with large outbreaks in poultry, wild birds, and wild mammals in Brazil, Argentina, and Uruguay, occurring during 2023 [18,71,85,86]. First detections of HPAIVs of clade 2.3.4.4b in wild aquatic birds in the Antarctic and sub-Antarctic regions of Falkland Islands, South Georgia, and South Sandwich Islands, were reported from September to December 2023, indicating the HPAI spread to new bird population groups [87,88]. 

**Figure 2:**
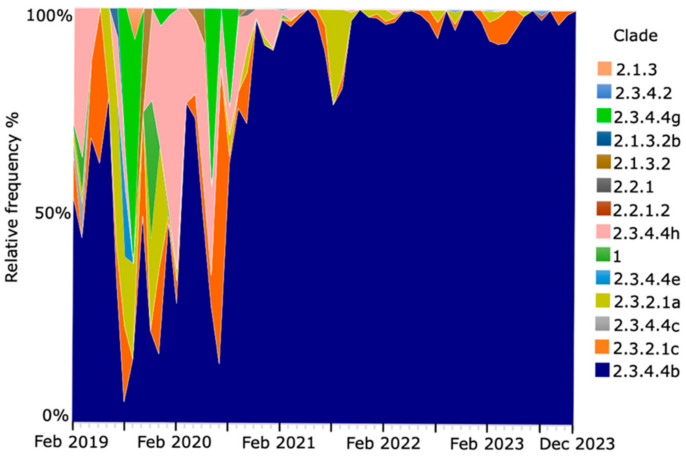
Relative frequency (%) over time of HPAI H5 Gs/Gd virus clade global isolations from February 2019 to December 2023. Based on data from GISAID’s EpiFlu Database and reproduced on Microsoft Excel (version 16.84) [92].

Sequence data submitted to the GISAID Epi-Flu Database (https://gisaid.org/about-us/acknowledgements/epiflu/, accessed on 11 January 2024) demonstrates the global dominance of HPAI H5Nx viruses of clade 2.3.4.4b that have emerged over time (Figure 2). With respect to the other 2.3.4.4 virus clades, H5N6 subtype of 2.3.4.4d-h clades has been predominantly isolated in China since 2014, and the geographic distribution of these isolations has been limited to East Asia [89,90,91]. Clade 2.3.4.4 H5N6 virus has only been detected in Asia, where it has circulated endemically since 2013, and distant geographical spread to other regions has not been reported yet [89].

The H5 clade 2.3.4.4b viruses have exhibited novel epidemiological and pathobiological characteristics distinct from other clades. An elevated pathogenicity has been observed in several duck species [93,94], considered as the AIV natural hosts, accompanied by asymptomatic infections in certain dabbling ducks [95,96,97]. Frequent die-offs have been observed, particularly in colony-breeding seabirds [98,99,100,101], which have been attributed to a shift in the HPAI virus epidemiology among waterbirds, resulting in the endemic circulation of these strains [102,103]. Due to its sustained circulation in the free-ranging avifauna, HPAI H5N1 virus is therefore considered as a new threat to wild bird conservation [104]. Globally threatened species, as defined by the IUCN Red List version 2022-2 [105], have been already decimated due to the infection, such as the above-mentioned seabirds [83], cranes (the hooded cranes, *Grus monacha*; the white-naped craned, *Grus vipio,* and red-crowned crane, *Grus japonensis*), Dalmatian pelicans (*Pelicanus crispus*), Bald eagles (*Haliaeetus leucocephalus*), and California condors (*Gymnogyps californianus*) [106,107,108,109]. With respect to California condors, given the impact of HPAIV H5N1-related mortalities on this critically endangered vulture, the emergency use of a vaccine to prevent additional deaths of these birds has been authorized by government authorities [110]. Detection of an HPAI H5N1 virus of the unknown clade in dead wild bird carcasses in the Galápagos islands has recently raised concerns for the conservation of endemic species such as the Galápagos lava gull (*Leucophaeus fuliginosus*) and the Galápagos penguin (*Spheniscus mendiculusi*) [111].

In the context of poultry, variable and even relatively lower mortality rates have been documented in experimental or natural infections with clade 2.3.4.4b viruses [112,113], with transmissibility rates varying within infected flocks [114,115]. Notably, certain HPAI H5N1 clade 2.3.4.4b virus genotypes, especially circulating in wild birds, have been associated with increased frequency of infections in non-human mammals [69,116], raising concerns for public health due to the identification of molecular markers of virus adaptation to mammalian hosts [117,118]. 

## 3. Wild Birds

### 3.1. Anseriformes Order

Within the Anseriformes order (ducks, geese, and swans), HPAI H5Nx viruses of clade 2.3.4.4b have been linked to a diverse range of infection outcomes and a variety of clinical and pathological manifestations, depending on the viral strain, the species considered, and the age of the infected birds. Experimental challenges of waterbird species with H5 clade 2.3.4.4b viruses are synthesized in Table 1. Data on domesticated Pekin ducks, which are equivalents to mallards [119], are reported in Table 2 under the domestic poultry section.

Dabbling ducks (*Anatidae*, Anatinae subfamily) have conventionally been regarded as more resistant than gallinaceous birds to overt HPAI clinical disease while maintaining high susceptibility to infection [126,127]. Notably, a sustained viral circulation in these species has been detected by surveillance efforts [68,116]. Among these, the mallard (*Anas platyrhynchos*) stands out as the most abundant and widely distributed duck species, serving as a crucial reservoir for LPAIVs [128]. Mallards’ higher resistance to HPAIV disease may be partially explained by the differences in cell signaling pathways compared to gallinaceous birds. Mallards possess RIG-I signaling pathways, which receptor-mediate IFN-β signaling in infected cells and the rapid induction of interferon-stimulated genes (ISGs) that critically limit HPAI virus spread and viremia [129,130]. However, experimental infections of mallards with HPAI viruses of clade 2.3.4.4b have exhibited an array of clinical signs, contingent upon the specific strain and the dose used for challenging the birds, as well as the age of the animals. Little to no clinical signs and a low to absent mortality rate were found in mallards infected with a 2017-H5N6 clade 2.3.4.4b virus isolated in Japan, namely A/mute swan/Shimane/3211A002/2017 [121]. However, when considering experimental infections with HPAI H5Nx viruses of clade 2.3.4.4, increased pathogenicity has been assessed for ducks infected with clade 2.3.4.4b viruses circulating in Europe during 2016–2017, in comparison to viruses of clade 2.3.4.4c [123,131]. This finding has been associated with differences in the translated amino acids in the viral genome, including internal genes, which confers novel pathobiological characteristics to the virus [132,133]. In a recent study, the synergic effect of HA, NP, NS, and, to a lesser extent, NA proteins contributed to the increased receptor binding affinity, sialidase activity, interferon antagonism, and replication that conferred heightened virulence in H5N8 HPAIV of clade 2.3.4.4b-infected mallards, in comparison to H5N8 HPAIV of clade 2.3.4.4c [134]. 

A spectrum of clinical signs can be observed during experimental infections with 2016-H5N8 clade 2.3.4.4b HPAIVs in mallards or Pekin ducks. These signs range from mild non-specific indications of HPAI infection (ruffled feathers, drooped wings, lethargy, and inappetence) to neurological signs, with a mortality rate ranging from 0 up to 80% depending on the strain used [94,96,123,135]. A notable weight loss has been associated with intrachoanal experimental inoculation of mallards with 10^7^TCID_50_/mL of A /Eurasian Wigeon/NL/4/2016 (H5N8) or 10^7^EID_50_/mL of A/Tufted-duck/Denmark/11470/LWPL/2016 (H5N8), compared to sham-inoculated control groups [94,122], with a dose-dependent effect [94]. Post-mortem examination (PME) of mallards experimentally infected with 2016-H5N8 HPAIVs of clade 2.3.4.4b revealed microscopic lesions similar to those described with other Gs/Gd lineage H5N1 HPAI viruses, such as congestion, petechial hemorrhages and/or ecchymoses, and necrotizing lesions in the brain and visceral organs [94,96,122,131]. Microscopic lesions associated with the infection differed according to the day of sampling [96]. At 4 days post-infection (dpi), multifocal mild to moderate lymphohistiocytic infiltrations and, rarely, mild parenchymal necrosis were observed in the myocardium, liver, and brain, where also multifocal gliosis was noticed. Lastly, the immunohistochemistry (IHC) results indicated the predilection of H5N8 for nervous tissue, myocardium, respiratory epithelium, and hepatic and pancreatic cells [96]. Virus shedding was predominantly linked to the respiratory route [93,122,131,135], consistent with previous experimental infections of ducks with other HPAIV clades [62,136,137,138,139], but in contrast to LPAIVs preferential replication in the gastrointestinal tract of ducks [140,141]. However, heightened cloacal shedding was observed during experimental infection of specific pathogen-free (SPF) Pekin ducks with intranasal and intratracheal inoculums of 10^7^ EID_50_/mL of clade 2.3.4.4b viruses circulating in Europe during 2016–2017, namely A/duck/Neth/16014829-001005/2016 and A/duck/Neth/17017236-001005/2017, in comparison to 2014-H5N8 HPAIV [123]. The phenomenon of “*increased enterotropism*” within recently circulating clade 2.3.4.4b viruses has been supported by immunohistochemical analysis in naturally infected individuals [142,143]. Virus antigen was detected in both the digestive and respiratory tracts of 2016-H5N8 and 2020-H5N8 HPAIV-infected wild ducks, as well as in the brain, liver, heart, and pancreas, in conjunction with necrosis and inflammation [143]. In the context of histopathological examination of naturally infected mallards with different subtypes of clade 2.3.4.4b viruses, the H5N1 HPAIV infection was linked to severe pancreatic necrosis [144], while H5N8-infected ducks, on the contrary, rarely exhibited mild pancreatic necrosis [145]. 

Despite frequent isolation of the virus in hunted, deceased, or moribund mallards during the 2021–2023 HPAI epidemics in Europe, the incidence of cases identified through passive surveillance (i.e., testing deceased or critically ill birds) has been lower in comparison to other waterbird species such as geese, swans, or terns. Historically, naïve geese and swans have been proven to be more susceptible to Gs/Gd H5N1 HPAIV infection and disease than mallards [116,146,147,148]. Regardless, pre-exposure to homologous or heterologous AIVs may also modulate the disease outcome. This has been previously demonstrated for mallards and wood ducks (*Aix sponsa*) challenged with other Gs/Gd strains isolated during 2005 and belonging to clade 2.2 [149,150]. Pre-exposure to homo- or heterosubtypic LPAIVs or HPAIVs resulted in protection against experimental HPAI virus challenge with 2.3.4.4b viruses, with a negligible infectious virus shedding from either the pharynx or cloaca insufficient to sustain a chain of virus transmission [93,96,122]. Following a less-virulent HPAIV challenge in wild ducks, long-term immunity (>one year) has been assessed [122]. Absent or mild microscopic lesions (mild lymphoplasmatic infiltrates in the liver, heart, muscle, and proventricular mucosa) were observed in birds pre-exposed to LPAIVs or HPAIVs [93,96,122]. Considering the protective effect of an extant immunity against HPAI infection, apparently healthy but actively infected mallards could potentially disseminate the virus and contribute to local or long-scale environmental contamination [96]. 

Although HPAI H5N8-infected satellite-tracked mallards were still capable of migration, suggesting a potential wide dispersion of the virus [151], other species of dabbling ducks are regarded as efficient long-distance carriers of HPAI viruses along migratory routes. The initial evidence of this was presented with the isolation of H5N8 HPAI viruses of clade 2.3.4.4c in fecal samples collected from healthy Eurasian wigeons (*Mareca penelope*) during the years 2014 and 2015 in the Netherlands [152,153], as well as in Russia [154]. Subsequently, during the period of active AIV surveillance in Italy from 2020 to 2021, Eurasian wigeons sampled and examined displayed no apparent clinical signs despite ongoing infections with HPAI H5Nx viruses of clade 2.3.4.4b. These infected wigeons exhibited active viral shedding through both the oropharyngeal and cloacal routes [97]. However, when subjected to controlled experimental infections with clade 2.3.4.4b viruses, namely A/duck/Neth/16014829-001005/2016 (H5N8) or A/duck/Neth/17017236-001005/2017 (H5N6) inoculated intranasally and intratracheally at a dose of 10^6^ EID_50_/mL, listlessness, ruffled feathers, reduced appetite, diarrhea, nasal discharge, and ultimately, neurological signs were observed and a notable mortality rate of 20% for H5N8-challenged individuals and 90% for H5N6-challenged individuals was recorded [123]. This was in line with the heightened pathogenicity of 2016–2017 HPAI viruses of clade 2.3.4.4b in ducks, in comparison with milder clade 2.3.4.4c infections [123,155]. In Europe, during the HPAI epidemic season of 2016–2017, it was estimated that up to 5% of the wintering population of Eurasian wigeons in the Netherlands might have succumbed to H5N8 virus circulation among wild birds [98]. Regarding HPAI virus shedding in Eurasian wigeons, an increased cloacal shedding was assessed for clade 2.3.4.4b viruses in experimentally inoculated individuals, in comparison to clade 2.3.4.4c virus inoculation [123]. Interestingly, IHC results of naturally infected individuals with HPAI H5N8 viruses circulating in Europe during 2016 confirmed virus replication in the intestinal tract [143]. However, in the case of individuals infected with the 2020-H5N8 HPAI virus strain, an intermediate level of virus attachment to intestinal epithelia was observed, suggesting incompatibility with virus replication at this site. Instead, these infected individuals displayed a high level of neurotropism, along with multifocal encephalitis characterized by areas of gliosis, neuronal degeneration, and necrosis [143]. 

Among dabbling ducks, Eurasian teals (*Anas crecca*) have also been proposed as potential long-distance vectors of HPAI infections due to asymptomatic disease [97,155,156]. Experimental infection of common teals with HPAI H5 2.3.4.4b viruses isolated in Japan during 2017, namely A/mute swan/Shimane/3211A002/2017 (H5N8), resulted in prolonged tracheal shedding and non-detectable clinical signs [124]. 

Turning attention to diving ducks (Anatidae, Aythyinae, Oxyurinae, and Merginae subfamilies), these inhabit freshwater, brackish, and coastal wetlands, often sharing ecological niches with dabbling ducks. Diving ducks are susceptible to AIV infection, yet limited reports have been published due to research and surveillance activities primarily focusing on dabbling ducks [157,158,159,160,161,162,163]. Following HPAI H5N8 of 2.3.4.4b clade natural infection, a significant number of tufted ducks (*Aythya fuligula*) was found to be deceased in Germany, displaying macroscopic changes such as severe hepatic necrosis, multifocal petechiae, and varying degrees of lung hyperemia and edema [164]. IHC examination revealed the presence of AIV nucleoprotein (NP) antigen associated with necrotic lesions in the liver, heart, brain, spleen, pancreas, and thymus [164]. However, a different infection outcome is suspected to be influenced by the bird’s immunological status. Similar to mallards, tufted ducks previously challenged with a less-virulent HPAIV (2014-H5N8 virus of clade 2.3.4.4c) before exposure to a more virulent 2016-HPAI H5N8 virus of clade 2.3.4.4b remained asymptomatic, avoiding body weight loss and mortality, in contrast to immunologically naïve control individuals directly challenged with the 2016-H5N8 [122]. Depending on the clade virus considered, HPAIV infection can, however, manifest different levels of pathogenicity. For instance, experimentally challenged common pochards (*Aythya ferina*) with an H5N8 HPAI clade 2.3.4.4c isolate from Europe (A/chicken/ Netherlands/emc-3/2014) became infected without evidence of disease, primarily shedding the virus through the pharynx [155]. These findings contrast with previous observations from experimental inoculation with an HPAI H5N1 virus of clade 2.2.1, which resulted in labored breathing, increased recumbency, neurologic signs, and mortality within 4 dpi [165]. Adults and juvenile ruddy ducks (*Oxyura jamaicensis*) and lesser scaups (*Aythya affinis*) were susceptible to experimental infection with HPAI H5N2 and H5N8 viruses of clade 2.3.4.4c obtained during 2014 [166]. Age-associated differences in clinical outcomes were observed, with higher disease susceptibility noted in juvenile ruddy ducks. Absence of clinical disease in adult ruddy ducks and lesser scaups suggested their potential reservoir role. However, low virus shedding, and a short duration of shedding indicated the inefficient role of these diving ducks in maintaining and disseminating the virus [166].

Geese and swans (Anatidae, Anserinae subfamily) are highly susceptible to HPAI clade 2.3.4.4b infection, with wild populations experiencing severe impact [70,167,168,169]. IHC results from naturally infected graylag goose (*Anser anser*) revealed 2016-HPAI H5N8 virus of clade 2.3.4.4b virus replication not only within the respiratory tract but also in the intestinal epithelium of deceased individuals [142]. Grossly, the primary pathological changes attributed to HPAIV H5Nx natural infection encompassed multifocal necrosis in the liver and pancreas, pin-point hemorrhages in the brain, sub-pericardial hemorrhages, and multifocal lung hemorrhages [143]. Notably, gross findings in H5N8-infected black swans (*Cygnus atratus*) were scarcely undetectable, with occasional mild pancreatic or splenic necrosis [145]. Hydropericardium was identified in a mute swan (*Cygnus olor*) that underwent necropsy and tested positive for HPAI H5N1 clade 2.3.4.4b virus through rRT-PCR detection [144]. As reported by Floyd et al. [170], in cases of HPAI H5N8 clade 2.3.4.4b virus infection in wild mute swans, gross findings were associated with liver and epicardial petechiae in one instance, and air sac opacity in another, contingent on the individual examined. Microscopic examination of tissues from one bird unveiled multifocal, necrotizing, nonsuppurative myocarditis, hepatitis, splenitis, nephritis, and encephalitis, accompanied by the intralesional presence of influenza A virus antigen, as observed in IHC analysis [170].

### 3.2. Charadriiformes Order

Gulls and other members of the Laridae family, within the Charadriiformes order, have exhibited a marked susceptibility to HPAI H5Nx viruses of clade 2.3.4.4b infection. Instances of mass mortality among adult seabirds have been documented across Europe, America, and Africa [81,101,171,172,173]. This underscores the rapid dissemination of infection attributed to their colony-breeding behaviors and scavenging tendencies. In the context of experimental infection, 8-week-old herring gulls (*Larus argentatus*) challenged with an HPAI H5N8 virus of clade 2.3.4.4b at 10^7^EID50/mL intraocular and intranasal inoculation (A/herring gull/Poland/MB082B/2016 (H5N8)) displayed a swift and severe onset of clinical signs as early as 24 h post-infection [125]. These signs encompassed heightened body temperature, depression, recumbency, reduced appetite, opisthotonos, torticollis, head tremors, paralysis, conjunctivitis, dyspnea, and nystagmus, alongside episodes of diarrhea. Within the 2–7 dpi period, 11 out of 12 directly infected gulls succumbed or were humanely euthanized due to the high mortality rate associated with the infection [125]. Similar to ducks, a greater survival rate was noted when birds had prior exposure to LPAI H5N1 virus infection. However, differently from ducks, pre-exposed birds exhibited abundant viral shedding from both the oral cavity and cloaca. PME subsequent to H5N8 experimental infection encompassed necrotizing inflammation of tissues, internal organ congestion (primarily intestines and lungs), and hemorrhages in subcutaneous tissues across the head, cerebral hemispheres, proventriculus, bursa of Fabricius, kidneys, liver, and spleen [125]. 

Natural HPAI H5N1 clade 2.3.4.4b infection in several species of terns, such as sandwich terns (*Thalasseus sandvicensis*), swift terns (*Thalasseus bergii*), common terns (*Sterna hirundo*), resulted in instances of die-offs [172,174,175]. A report from the Netherlands reported that afflicted sandwich terns exhibited marked debilitation, rendering them unable to take flight and predominantly lethargic; some were observed with wings outstretched. In later stages of the illness, certain individuals presented with opisthotonos and, on occasion, flipped over backwards. Among the four adult birds subjected to necropsy and subsequently confirmed by PCR analysis, IHC results revealed viral antigen expression in the pancreas (N = 3), duodenum (N = 4), or lung and nasal tissue (N = 1), colocalized with necrosis and inflammation [174]. 

In July 2021, in Scotland, notable die-offs of great skuas (*Stercorarius skua*) were observed, with instances escalating to the extent that mass mortality events were recorded among various breeding populations [176]. Birds submitted for PME were generally in good physical condition, occasionally displaying vent soiling or an empty proventriculus and gizzard. Histologically, the frequently encountered changes associated with HPAI H5N1 infection encompassed severe pancreatic necrosis, along with mild to moderate meningoencephalitis. In rare instances, findings included myocardial necrosis, moderate adrenal necrosis, mild hepatic necrosis, and proventricular necrosis. During the summer of 2022, an additional decrease in the number of breeding colonies of great skuas was observed in the Shetland islands of the UK, coinciding with an unusually high occurrence of deceased individuals attributed to HPAI H5N1 clade 2.3.4.4b infection [177]. While PME or histopathological analyses were not conducted, a comprehensive description of clinical manifestations was provided. Affected individuals exhibited distinct neurological symptoms, including walking in circular patterns, stumbling over their own feet, rolling movements, and a drooping head. As their condition deteriorated and they succumbed, these birds demonstrated severe hyperextension and spasticity in the head and neck region [177].

Within the Charadriiformes order, HPAIVs of clade 2.3.4.4b have also been detected in various species of wild shorebirds [54]. These hold a central role in the ecology of LPAIVs in North America [178], though they are less frequently sampled during HPAI surveillance efforts in Europe [99,100]. To date, experimental infections involving HPAI H5 viruses of clade 2.3.4.4b have yet to be published for these birds. However, a study conducted by Wille et al. [179] reported the presence of antibodies against this H5 clade in red-necked stints (*Calidris ruficollis*) sampled during the 2016–2017 austral summer, thereby indicating prior virus exposure in this long-distance migratory wader.

### 3.3. Other Aquatic Wild Birds

The incursions of HPAI H5Nx viruses of clade 2.3.4.4b have also impacted African wild birds, particularly those in South and West Africa, owing to the presence of wetlands that host substantial numbers of potentially infected migratory birds from Eurasia during the winter months [180]. In January 2019, notable mortalities were documented among African penguins (*Spheniscus demersus*), an endangered penguin species, in Namibia due to HPAI H5N8 infection [181]. Prior to demise, afflicted individuals exhibited symptoms such as emaciation, torticollis, twitching, incoordination, corneal opacity, and lethargic or comatose behavior. Upon post-mortem examination, the carcasses appeared externally normal, though a grayish-greenish soiling of the peri-cloacal region was observed in two cases. Necropsy findings unveiled fibrinous strands within the coelomic cavity, widespread visceral congestion, and segmental hemorrhages along the gut in all instances. Instances of mass mortalities among African penguins due to H5N8 HPAIV infection had been previously documented in the Western and Eastern Cape Provinces of South Africa, alongside other migratory aquatic species, including Cape gannets (*Sula capensis*) and Cape cormorants (*Phalacrocorax capensis*) [175], in the absence of further clinic-pathological examination. Additionally, Cape cormorants, an endangered bird species endemic to the southwestern African coasts, recently experienced HPAI H5N1 clade 2.3.4.4b outbreaks, resulting in a significant decline in a Namibian colony in 2022 [182]. Lastly, great white pelicans (*Pelecanus onocrotalus*) infected with HPAIV H5N1 displayed paralysis, dyspnea, diarrhea, and subcutaneous hemorrhages [183]. 

### 3.4. Game Birds

Minor gallinaceous species have been recognized as susceptible to HPAIV infection, although they have been rarely investigated in comparison to domestic poultry or wild aquatic birds [184]. Furthermore, game birds have been considered as potential bridge hosts for AIV incursion in poultry farms [184]. In the context of HPAI H5 clade 2.3.4.4b, the literature offers scant reports. Wild ring-necked pheasants (*Phasianus colchicus*) have been found to be HPAIV of clade 2.3.4.4b-infected in several European countries, demonstrating systemic virus tropism resembling that in chickens with high morbidity and mortality [54,185]. Notably, in Finland, mass mortality events of pheasants released on hunting grounds led to the spillover of an HPAI H5N1 virus of clade 2.3.4.4b that severely affected wild carnivores [186]. Intranasal and intraocular experimental infections of pheasants with different doses (10^5^ or 10^7^ EID_50_/mL) of A/pheasant/Denmark/12106–3/2018, an H5N8 virus of clade 2.3.4.4b showed evident clinical signs (drooped wings, huddling, ruffled feathers and lethargy, associated to neurological signs in some individuals), up to 100% mortality and efficient transmission to naïve pheasant or chicken contact birds, confirming the role of pheasants as bridging hosts for the infection of commercial poultry [187]. Similarly to experimental infection, naturally infected pheasants with 2017-HPAI viruses of clade 2.3.4.4b displayed severe clinical signs with neurological disorders [188,189]. PME findings aligned with prior observations in other gallinaceous species, characterized by necrotic and hemorrhagic lesions in visceral organs [184,188,189]. However, a novel and consistent discovery was the identification of diphtheroid plaques in the oropharyngeal mucosa linked to viral-induced necrotizing stomatitis of the epithelium [188]. IHC results demonstrated viral antigens consistently detected in parenchymal and endothelial cells within the encephalon, heart, and nasal mucosa and less frequently in the kidney, pancreas, and liver. PCR results indicated a higher virus shedding via the oropharynx [188].

Evidence from necropsies on naturally HPAIV H5N1 clade 2.3.4.4b-infected pheasants, peafowl (*Pavo cristatus*), and guinea fowls (*Numida meleagris*) in the UK has primarily revealed pancreatic and splenic necrosis, accompanied by hydropericardium and epicardial petechiae [144]. 

### 3.5. Pigeons and Doves

Pigeons and doves are susceptible to HPAI infection; however, they do not usually exhibit clinical signs and are considered ineffective vectors of the virus due to low viral shedding [190]. Nevertheless, varying outcomes of infection can be observed depending on the viral clade used for experimental infection [90,191]. Natural infections of H5N8 HPAIVs of clade 2.3.4.4b in wild columbids in South Africa during 2017 resulted in atypical die-offs, possibly exacerbated by concurrent avian orthoavulavirus infection [175]. A noteworthy instance of natural HPAI H5N1 clade 2.3.4.4b infection was documented in a wood pigeon (*Columba palumbus*) that was found dead in a German wildlife sanctuary [192]. Gross findings included mild enlargement of the spleen and a few greyish discolorations in the pancreas, mild splenic enlargement, and a few greyish discolorations in the pancreas. Histologically, acute lymphohistiocytic meningoencephalitis was observed, accompanied by mild perivascular cuffing and neuronal necrosis in the grey matter of the cerebral hemispheres and brain stem. Additionally, the pancreas displayed severe multifocal to coalescing necrotizing pancreatitis. IHC results unveiled a pronounced neurotropism of the virus [192].

### 3.6. Scavengers and Raptors

Due to their feeding ecology, scavengers (such as corvids, nocturnal and diurnal raptors) and birds of prey (falcons, hawks, eagles, owls) have the potential to come into contact with AIV-infected prey or carcasses [193]. Consequently, HPAI epizootics involving clade 2.3.4.4b viruses have had severe impacts on these bird populations [70,98,194,195]. In the context of the Accipitriformes order, natural HPAI H5N1 virus of clade 2.3.4.4b infection in an African fish eagle (*Icthyophaga vocifer*) resulted in gross findings of carcass dehydration and edematous brain [196]. In 2017, die-offs in white-tailed eagles (*Haliaeetus albicilla*) were attributed to H5N8 clade 2.3.4.4b infection [194]. Infected birds found alive exhibited neurological symptoms, including torticollis, opisthotonus, limber neck, ataxia, and circling movements. PME revealed scant or absent gross lesions typically associated with HPAI. However, histopathological and immunohistological investigations unveiled oligo- to multifocally necrotizing lesions in the cerebrum, cerebellum, and brain stem. Immunohistological and virological analyses confirmed the presence of influenza A virus NP antigen and/or H5N8-specific RNA in organ samples [194]. PMEs of HPAI H5N8-naturally infected common buzzards (*Buteo buteo*) found in the Netherlands did not reveal any external lesions, with birds in a moderate to good state of nutrition, highlighting the acute nature of their deaths [197]. Among the 11 birds examined, only 2 displayed gross abnormalities, including well-delimited, multifocal hemorrhages in the heart and brain of one individual and a swollen liver with a rounded margin in another. Multifocal encephalitis with foci of gliosis, neuronal degeneration, and necrosis was reported in the majority of the birds, indicating the neurotropic nature of the viral strain. Furthermore, the highest amount of viruses based on PCR results was detected in the myocardium, indicating the cardio-tropism of the strain, with focal and extensive myocardial necrosis observed. Interestingly, necrotic lesions were found in the proventriculus of two common buzzards, with viral antigens present in the epithelial cells [142]. 

Among corvids (Passeriformes order), virus histochemistry revealed the presence of HPAI H5N8 virus antigen in both the respiratory and digestive tract of naturally deceased Eurasian magpies (*Pica pica*) [142], which are often found around poultry houses and have been regarded as bridge hosts for avian influenza transmission between domestic and wild birds [193].

## 4. Poultry

HPAIV infection in chickens and turkeys is typically associated with high morbidity and mortality. Clinical signs manifest as systemic virus replication and damage to visceral organs, as well as cardiovascular and nervous systems [198]. However, a fulminating disease with sudden death is also frequently observed. HPAI-infected chickens have an early and increased production of proinflammatory molecules and succumb to cytokine storm [199,200,201]. Gross lesions can vary based on factors such as the virus strain, the duration between infection and death, and the age and species/breed of the poultry affected [13]. Regarding HPAIV H5Nx of clade 2.3.4.4b, both experimental and natural infections have been documented in major poultry species. An overview of the experimental challenges involving HPAIV viruses of clade 2.3.4.4b hereby gathered is reported in Table 2. 

**Table 2:** Selected studies reporting experimental infections with highly pathogenic avian influenza (HPAI) H5Nx clade 2.3.4.4b strains in domesticated poultry species.

Species	Age	Virus	Route of Infection (Dose)	Morbidity * (%)	Mortality (%)	S or A	MDT	Transmission	Ref.
Pekin duck	1 wk	A/tufted duck/ Germany/AR8444-L01987/2016 (H5N8)	IM ^1^ (10^6^TCID_50_/bird)	10/10 (100)	10/10 (100)	S	2	Yes	[131]
	5–6 wk	A/wigeon/Wales/052833/2016 (H5N8)	IN and IO (10^7^EID_50_/mL)	8/8 (100)	0/8 (0)	A	n.a.	Yes	[135]
			IO and IN (10^5^EID_50_/mL)	5/5 (100)	1/5 (20)	S	9.13	Yes	
			IO and IN (10^3^EID_50_/mL)	5/5 (100)	2/5 (40)	S	4.42 and 9.13	Yes	
	7 wk	A/duck/Neth/16014829-001005/2016 (H5N8)	IN and IT (10^7^EID_50_/mL)	10/10 (100)	0/10 (0)	A	n.a.	n.a.	[123]
		A/duck/Neth/17017236-001005/2017 (H5N6)	IN and IT (10^7^EID_50_/mL)	10/10 (100)	6/10 (60)	S	≥3	n.a.	
	14 mo	A/tufted duck/Germany/AR8444L01987/2016 (H5N8)	IN (10^6^EID_50_/mL)	10/10 (100)	10/10 (100)	S	1–8	Yes	[93]
	adult	A/tufted duck/ Germany/AR8444-L01987/2016 (H5N8)	IO and IN (10^6^TCID_50_/bird)	10/10 (100)	2/10 (20)	A	4–5	Yes	[131]
Domestic duck	2 wk	A/mandarin duck/ Korea/H242/2020 (H5N8)	IN (10^7^EID_50_/mL)	5/5 (100)	0/5 (0)	A	n.a.	Yes	[202]
		A/duck/Korea/HD1/2017 (H5N6)	IN (10^7^EID_50_/mL)	5/5 (100)	0/5 (0)	A	n.a.	Yes	
Muscovy duck	1 wk	A/tufted duck/ Germany/AR8444-L01987/2016 (H5N8)	IM ^1^ (10^6^TCID_50_/bird)	10/10 (100)	10/10 (100)	S	≥2	n.a.	[131]
Chicken–Ross 308	2 wk	A/Goose/Spain/ IA17CR02699/2017 (H5N8)	IN (10^5^ELD_50_/0.05mL)	15/15 (100)	1/15 (8)	S	6	n.a.	[203]
Chicken–White Leghorn			IN (10^5^ELD_50_/0.05mL)	13/13 (100)	3/13 (25)	S	3.7	n.a.	
	4 wk	A/common-coot/Egypt/CA285/2016 (H5N8)	IN (10^7^EID_50_/mL)	10/10 (100)	10/10 (100)	S	8.1	Yes	[204]
		A/duck/Egypt/F446/2017 (H5N8)	IN (10^7^EID_50_/mL)	10/10 (100)	10/10 (100)	S	2.5	Yes	
		A/duck/Egypt/SS19/2017 (H5N8)	IN (10^7^EID_50_/mL)	10/10 (100)	10/10 (100)	S	4.2	Yes	
		A/American Wigeon/South Carolina/22-000345-001/2021 (H5N1)	IC (10^3.6^EID_50_/mL)	3/5 (60)	3/5 (60)	S	2.7	Yes	[205]
			IC (10^5.6^EID_50_/mL)	5/5 (100)	5/5 (100)	S	2	Yes	
			IC (10^7.6^EID_50_/mL)	5/5 (100)	5/5 (100)	S	1	Yes	
		A/Tufted duck/Denmark/11740-LWPL/2016 (H5N8)	IC (10^1.5^EID_50_/mL)	0/5 (0)	0/5 (0)	A	n.a.	No	
			IC (10^2.8^EID_50_/mL)	1/5 (20)	1/5 (20)	S	3	No	
			IC (10^6.7^EID_50_/mL)	5/5 (100)	5/5 (100)	S	2	Yes	
	5 wk	A/mandarin duck/ Korea/H242/2020 (H5N8)	IN (10^7^EID_50_/mL)	5/5 (100)	5/5 (100)	S	4.3	Yes	[202]
		A/duck/Korea/HD1/2017 (H5N6)	IN (10^7^EID_50_/mL)	5/5 (100)	5/5 (100)	S	2.2	Yes	
	6 wk	A/tufted duck/ Germany/AR8444-L01987/2016 (H5N8)	IV ^2^ (n.d.)	10/10 (100)	10/10 (100)	S	2	Yes	[131]
		A/domestic duck/ Siberia/49feather/2016 (H5N8)	IV ^2^ (10^7^EID_50_/mL)	10/10 (100)	10/10 (100)	S	3	n.a.	[206]
		A/tufted-duck/ Denmark/11740/ 2016 (H5N8)	IC (10^7^EID_50_/mL)	13/13 (100)	13/13 (100)	S	2.1	No	[115]
		A/turkey/Hungary/53433/2016 (H5N8)	IC (10^7^EID_50_/mL)	13/13 (100)	13/13 (100)	S	2.1	No	
Turkey–Commercial BBW	3 wk	A/American Wigeon/South Carolina/22-000345-001/2021 (H5N1)	IC (10^3.6^EID_50_/mL)	4/5 (80)	5/5 (100)	S	4.6	Yes	[205]
			IC (10^5.6^EID_50_/mL)	5/5 (100)	5/5 (100)	S	3.4	Yes	
			IC (10^7.6^EID_50_/mL)	5/5 (100)	5/5 (100)	S	2.6	Yes	
		A/Tufted duck/Denmark/11740-LWPL/2016 (H5N8)	IC (10^1.5^EID_50_/mL)	0/5 (0)	0/5 (0)	A	n.a.	No	
			IC (10^2.8^EID_50_/mL)	0/5 (0)	0/5 (0)	A	n.a.	No	
			IC (10^6.7^EID_50_/mL)	5/5 (100)	5/5 (100)	S	3	Yes	
	5–6 wk	A/wigeon/Wales/052833/2016 (H5N8)	Contact with infected ducks	12/12 (100)	12/12 (100)	S	2.96 to 8.54	n.a.	[135]

S, symptomatic infection; A, asymptomatic infection; MDT, mean death time; IC, intrachoanal route; IN, intranasal route; IM, intramuscular route; IO, intraocular route; IT, intratracheal route; IV, intravenous route. n.a., not applicable. n.d., not defined. ^1^ Intramuscular pathogenicity index testing. ^2^ Intravenous pathogenicity index testing. * Infected individuals based on RT-PCR or virus isolation or HI assay.

### 4.1. Chickens

Chickens (*Gallus gallus domesticus*) are highly susceptible to HPAI, with the severity of infection varying depending on the viral strain and host factors (species, breed, age at infection, and immune response) [25,207,208]. The inoculation of most HPAIVs in chickens causes evident clinical signs (e.g., apathy, nervous signs) and gross lesions (e.g., cutaneous edema, cyanosis of the comb and wattles, hemorrhages in skin), and chickens usually die shortly after the infection [25,209]. 

With respect to 2016-HPAI viruses of clade 2.3.4.4b, SPF chickens intravenously infected with HPAI H5N8 strains, namely A/tufted_duck/Germany/AR8444L01987/2016 or A/domestic duck/Siberia/49feather/2016, showed systemic disease and rapid death within 2–3 dpi [131,206]. Classic lesions of HPAI infections were noticed in visceral organs, such as necrosis and necrotizing inflammation [131,206,210]. A pronounced neurotropism was noticed for A/domestic duck/Siberia/49feather/2016, with the highest titer observed in the brain (6.75 ± 0.07 log_10_TCID_50_/mL) [206]. Following the natural route of infection, though, HPAIVs H5Nx clade 2.3.4.4 have shown varying and relatively lower mortality rates in experimentally inoculated or contact-exposed chickens in comparison to previous HPAIVs H5N1 of the Gs/Gd lineage that caused almost 100% mortality [113,211,212]. In testing the pathogenicity and transmissibility of three H5N8 Egyptian isolates of clade 2.3.4.4b collected during 2016–2017, namely A/common-coot/Egypt/CA285/2016 (CA285), A/duck/Egypt/F446/2017 (F446) and A/duck/Egypt/SS19/2017 (SS19), in SPF chickens inoculated intranasally, a systemic viral replication and inflammatory and necrotizing changes in organs were noticed in all the infected groups [204]. Chickens challenged with the F446 strain exhibited higher transmissibility and pathogenicity (shorter mean death time, MDT) than CA285 and SS19, likely due to the presence of a more adapted reassorted polymerase complex. Higher viral titers in swabs and organs associated with a relatively delayed mortality (4–7 dpi MDT versus 3 dpi MDT) were noticed in comparison to an HPAIV H5N1 strain, namely A/chicken/Egypt/15S75/2015 (clade 2.2.1.2), used as a control [204]. Differently, a 100% mortality within 3 dpi was shown in SPF chickens inoculated with two HPAI H5N8 viruses of clade 2.3.4.4b isolated in Europe (A/turkey/Hungary/53433/2016 and A/tufted-duck/Denmark/11740/2016) [115]. Notably, a higher oropharyngeal viral shedding was observed compared to cloacal shedding, but no transmission to co-housed chickens occurred. Microscopic examination revealed necrosis of parenchymal cells, accompanied by lymphoplasmacytic or heterophilic inflammatory infiltrates, consistent with other groups infected with viruses belonging to 2.3.4.4a, 2.3.4.4c, 2.3.4.4d, and 2.3.4.4e H5 clades. Remarkably, virus replication was prominently evident in villi enterocytes and capillaries, often leading to focal areas of necrosis with lymphoplasmacytic infiltrates, similar to the findings seen in HPAI H5 2.3.4.4a and 2.3.4.4e clade virus-challenged individuals [115]. 

During the 2020–2021 HPAI H5Nx of clade 2.3.4.4b virus incursions in Europe, PMEs of chickens succumbed to infections reported frequent cyanosis of the combs or reddening of the feet, facial edema, and pancreatic and splenic necrosis [145]. Notably, 10.3% (24 out of 230) of outbreaks in broilers were associated with the absence of mortality and/or clinical signs [70]. This “*silent infection behavior*” was associated with minimal tracheal and/or cloacal viral shedding, and, considering the potential delayed outbreak suspicion and diagnosis on farms due to scarce clinical signs, additional sampling strategies have been proposed for these poultry species [69,112]. The lower pathogenicity of a 2020-HPAI H5N8 virus of clade 2.3.4.4b (A/mandarin duck/Korea/H242/2020) in comparison to a 2016-HPAI H5N6 virus of the same clade (A/duck/Korea/HD1/2017) has been assessed in five-week-old SPF chickens experimentally challenged [202]. The results revealed that chickens inoculated with the 2020-HPAI H5N8 virus exhibited a longer survival period, with an MDT of 4.3 days, compared to the 2.2 days observed for the 2016-HPAI H5N6 virus. Notably, a higher viral shedding titer from both oral and cloacal routes was detected through virus isolation of HPAIV H5N8 from swabs in comparison to the HPAI H5N6 strain. This finding suggests an increased risk of viral contamination on farms and delayed epidemic control [202]. 

A different pathogenicity of HPAIV infection can be observed with respect to the chicken breed/line considered [213,214,215]. SPF chickens and Ross 308 broilers were more resistant to HPAI experimental inoculation with A/Goose/Spain/IA17CR02699/2017 (H5N8) of clade 2.3.4.4b virus in comparison to four Spanish local breeds (Empordanesa, Penedesenca, Catalana del Prat, Flor d’Ametller, Euskal Oiloa) [203]. The more resistant chicken breeds displayed less frequency of severe clinical signs, lower mortality rates, and lower numbers of animals that shed the virus. This suggests that the genetic background may confer a higher natural resistance to different HPAIVs subtypes, in line with a previous report [216]. 

### 4.2. Turkeys

Turkeys (*Meleagris gallopavo*) are particularly susceptible to HPAI infection, often experiencing more severe impacts compared to other poultry species [12,217,218].

In relation to HPAIV strains of clade 2.3.4.4b circulating in Europe during the autumn of 2016, SPF turkeys in contact with Pekin ducks infected with different doses of A/wigeon/Wales/052833/2016 (H5N8) (10^7^, 10^5^, 10^3^, 10^1^ EID_50_/mL given intraocularly and intranasally) all became infected and died [135]. The longest MDT (8.54 days) was observed in turkeys exposed to the lowest duck-dose group, and the onset of clinical signs, such as loss of balance, inability to seek food or water, tremors, and diarrhea, ranged between 6 and 14 h prior to death. PME of dead or humanely euthanized turkeys revealed moderate splenomegaly and mottling. IHC results confirmed systemic infection and indicated the presence of AIV antigen in adrenal medulla ganglions, ependymal cells of the central nervous system (associated with pronounced lymphocytic meningitis and perivascular cuffing), thymus epithelia, and exocrine pancreas epithelia. Furthermore, IHC findings underscored the widespread distribution of the virus, including feather follicles, air sacs, thymus, liver, ovary/testes, nasal cavity, and gizzard [135]. PMEs in field cases of HPAI H5N8 and H5N1 virus infections in turkeys during the 2020–2021 outbreaks in the UK reported facial edema in a small number of individuals, particularly around the masseter, submandibular, periocular, or conjunctiva areas. A substantial proportion of infected turkeys exhibited pancreatic necrosis, often appearing as multifocal to coalescing tan discoloration and, in some instances, forming target-like lesions. All H5N8-infected turkeys exhibited enlarged and rounded spleens, featuring multifocal white mottling. Kidney lesions were present in 33% of the examined turkeys, characterized by swollen parenchyma displaying areas of pallor and occasional petechiae within the renal subcapsular spaces or peri-renal fat. Pancreatic and splenic necrosis were also commonly observed in HPAI H5N1-infected individuals [144]. In an experimental setting comparing HPAI H5N1 of clade 2.3.4.4b infection in chickens and turkeys, the latter exhibited longer MDT (2.6–8.2 days versus 1–4 days for chickens), increased virus shedding, and facilitated transmission to contact birds [205].

Following the introduction of the H5 clade 2.3.4.4b in North America in 2021 [72,73], Malmberg et al. [219] investigated the pathological effects of HPAI H5N1 virus infection in wild turkeys found deceased near an infected backyard poultry farm. External gross lesions commonly associated with HPAIV infection in poultry, such as cyanosis, facial edema, and petechiae, were not observed. Instead, the prevailing gross lesions included mild to marked, multifocal to coalescing necrosis of the ovary and oviduct, along with mottling of the spleen. Furthermore, moderate to marked diffuse edema and congestion of the lungs were noted. Upon histopathological examination, all examined individuals displayed signs of acute multiorgan necrosis in various organs, including the lungs, liver, gonads, and lymphoid tissues of the spleen and gastrointestinal tract. Notably, no significant lesions were identified in the heart or trachea. A lower number of individuals showed necrotic or inflammatory lesions in the brain, pancreas, kidney, esophagus, and thymus [219].

### 4.3. Domestic Ducks and Geese

Consistent with field observations in wild waterfowl, experimental studies using 2016–2016 HPAI H5 viruses of clade 2.3.4.4b in domestic duck breeds resulted in high virus replication and a range of mortality rates [94,131,135]. 

Severe disease was reported in Egyptian ducks from a meat duck farm during an HPAI H5N8 outbreak in 2017 in Egypt [220]. Clinical signs included ruffled feathers and nervous system involvement, such as abnormal head position, recumbency, and tremors, resulting in a 90% mortality rate. Gross observations revealed multifocal areas of encephalomalacia in the cerebrum, petechial hemorrhages on the cerebellar surface, and cerebellar swelling. Furthermore, the lung, kidney, and spleen displayed congestion, while the pancreas exhibited multifocal areas of necrosis [220]. Previously, similar lesions in the brain were reported in HPAI H5N2-, H5N6-, and H5N8 virus-infected domestic Pekin ducks [221] and H5N8-infected fattening ducks [222]. An increased neurovirulence of clade 2.3.4.4b virus infection [A/tufted_duck/Germany/AR8444L01987/2016 (H5N8)] in Pekin ducks inoculated by the oculo-nasal route was reported in comparison to 2.3.4.4c virus infection [(A/turkey/Germany-MV/R2472/2014 (H5N8)] [131]. Ducks dying following 2016-HPAIV H5N8 of clade 2.3.4.4b experimental infection consistently exhibited moderate, multifocal, acute to subacute, necrotizing polioencephalitis with intralesional antigen-positive neuroglial cells and high viral titer levels in the brain, comparable to those of lung samples of the same animals. In contrast, no inflammatory changes and no antigens were detectable in the brains of 2014-H5N8 clade 2.3.4.4c challenged ducks [131]. The predominant neurotropism of HPAI virus in domestic ducks has been linked to a combination of early colonization of the central nervous system (CNS) in HPAIV H5N8-experimentally challenged individuals and the prolonged survival after the onset of virus replication, allowing the virus and the host response to cause significant tissue damage to the brain tissue [223]. Genetic differences between HPAI-H5N8 viruses of clade 2.3.4.4c and 2.3.4.4b might influence the differences in duck pathogenesis, following continuous evolution through genetic reassortment [134,224]. A combination of virulence factors expressed by the HA, NP, NS, and, to a lesser extent, NA [225], and species-related factors (e.g., breed, age, route of inoculation, challenge dose) play a crucial role in the severity of the disease developed following HPAIV infection in ducks. Domestic ducks can also display absent clinical signs, prolonged viral shedding, and a 100% transmission rate to contact birds, as demonstrated in an experimental study involving A/mandarin duck/Korea/H242/2020 (H5N8) and A/duck/Korea/HD1/2017 (H5N6) intranasally inoculated at low to high doses (10^3^, 10^5^, and 10^7^EID_50_/mL) [202].

In the case of domestic geese, Zhu et al. [226] documented an HPAI H5N8 outbreak in January 2021 on an egg-laying farm in Northeastern China. The overall mortality rate reached 50%, with affected individuals displaying cyanosis in the head, sarcoma, oral mucosa, tongue, and hemorrhagic conjunctiva and nostrils. PMEs of the coelomic cavity revealed lesions primarily localized in the digestive and respiratory tracts, including hemorrhagic trachea, larynx, liver, and glandular gastric papillae. Additionally, a diffuse hemorrhage was observed in the intestine and pancreas [226]. Natural cases of HPAI H5N1 infections in domestic geese in the UK were associated with pancreatic lesions [144]. 

## 5. Mammals

Since 2016, HPAI H5N1 viruses of clade 2.3.4.4b have been associated with global spillover events involving wild and domestic mammals and, in rare instances, humans exposed to infected birds [117,227]. Among wild mammal species, the infection with HPAI H5Nx viruses has been most frequently detected in predators or scavengers such as wild canids, felids, and marine mammals that hunt infected wild birds and/or feed on wild bird carcasses. With respect to outbreaks of H5Nx in farmed mammals, these were linked to virus introduction via wild birds through direct or indirect contact [228,229,230] or through exposure to infected poultry [231]. Notably, instances of mass mortality in pheasants released in hunting estates in southern Finland have been attributed to virus infections in wild mammals, underscoring the significance of robust surveillance efforts in wildlife [186]. While conclusive evidence of mammal-to-mammal transmission remains elusive, die-off events among wild sea lions along the Pacific coast of South America, as well as outbreaks in fur farms across Europe, suggested the potential for avian-independent transmission of the virus [81,84,228,229]. 

Even pets have not been spared from HPAI virus of clade 2.3.4.4b infection. In Poland, domestic cats (*Felis catus*) were found to be HPAI H5N1-infected around mid-2023, likely as a result of consuming contaminated poultry meat [232]. In Italy, dogs (*Canis lupus familiaris*) and a cat were found to have seroconverted following exposure to the H5N1 virus during a poultry outbreak [117]. 

Since 2022, a small number of sporadic human infections with H5 HPAI have been reported worldwide. As of April 2024, thirteen (13) cases were associated with clade 2.3.4.4b HPAI A(H5N1) virus in seven countries (Cambodia, Chile, China, Ecuador, Spain, United Kingdom, United States), and eleven (11) cases were associated or assumed to be associated with clade 2.3.2.1c HPAI A(H5N1) viruses in Cambodia and Vietnam. Nearly all human cases of HPAIV H5 reported followed recent exposure to sick or dead poultry, and no evidence of human-to-human transmission was identified [59,233]. One single mild human case was associated with exposure to infected dairy herds in Texas. Clinical signs ranged from asymptomatic or mild illness [such as eye redness (conjunctivitis) or mild flu-like upper respiratory symptoms] to severe (such as pneumonia requiring hospitalization) and included fever (temperature of 100 °F [37.8 °C] or greater), cough, sore throat, runny or stuffed nose, muscle or body aches, headaches, fatigue, and shortness of breath or difficulty breathing. Less common signs and symptoms included diarrhea, nausea, vomiting, or seizures. Fourteen human cases (seven children and seven adults) reported severe or critical illness, and seven (three children and four adults) died [59]. Genetic data have revealed the acquisition of markers of mammalian adaptation in those strains that infected humans and certain mammals. This indicates that the virus may undergo intra-host evolution, resulting in genetic changes that allow more efficient replication in the lower respiratory tract or extrapulmonary tissues. Changes include the substitutions 591K, 627K, and 701N in the polymerase basic protein 2 (PB2) [234,235]. 

### 5.1. Farmed Animals and Pets

Among farmed animals, domestic pigs (*Sus scrofa*) have always been spotlighted in the context of AIV infection due to their role as a “*mixing vessel*” in the virus epidemiology, enabling further reassortment between human, avian, and swine strains [236]. Serological detections of HPAI H5Nx clade 2.3.4.4b exposure have been identified in asymptomatic pigs across various farms in France [230], as well as in healthy pigs sampled during an HPAI H5N1 outbreak on a rural mixed-species poultry farm in Italy [231]. From the literature to date, a single experimental challenge in pigs has been reported by Graaf et al. [237]. In this study, four-month-old pigs were nasally exposed to an HPAI H5N1 virus (A/chicken/Germany/AI04286/2022), yet no clinical manifestations or pathological changes were observed. Among the eight pigs, only one seroconverted at 14 dpi, and minimal levels of viral RNA were detected in the respiratory, alimentary, and brain tissues of this individual. The authors concluded that pigs are unlikely to serve as significant carriers for transmitting this specific genotype of HPAI virus H5N1 clade 2.3.4.4b among pigs and across interfaces [237]. 

As of April 2024, The U.S. Department of Agriculture (USDA) Animal and Plant Health Inspection Service (APHIS) has confirmed the detection of highly pathogenic avian influenza (HPAI) in goats in Minnesota (1) and dairy herds in Texas (12), Kansas (4), Michigan (6), New Mexico (6), Idaho (2), North Carolina (1), Ohio (1), South Dakota (1), and Colorado (1) [59,238]. Adult bovines presented decreased lactation, low appetite, and other non-specific clinical signs [23]. Affected dairy farms have reported about 10% morbidity of their milking cows, but there have been no confirmed deaths associated with HPAI in dairy cattle yet [239]. In addition, one person in Texas tested positive for HPAI A(H5N1) virus after exposure to the infected ruminants [240]. In Minnesota, detection was prompted by unusual deaths of newly kidded goats on the property where a backyard poultry flock had been depopulated due to HPAI in February 2024. The goats and poultry had access to the same areas and included a shared water source [241].

Different outcomes have been noted in farmed animals of the Carnivora order. Specifically, American minks (*Neovison vison*) raised for fur production in Spain exhibited a sudden increase in mortality rate (0.77% versus 0.2–0.3% during the initial phase of the outbreak) due to HPAI H5N1 infection [228]. Clinical signs included loss of appetite, hypersalivation, depression, bloody snouts, and neurological manifestations like ataxia and tremors. PME revealed either hemorrhagic pneumonia or red hepatization of the lungs. Elevated viral loads were detected through rRT-PCR analysis of oropharyngeal and rectal swabs. As the infection spread across the holding and affected more minks, onward transmission of the virus within the population was suspected [228]. Since mid-July 2023, HPAI H5N1 outbreaks have impacted American minks, raccoon dogs (*Nyctereutes procyonoides*), arctic foxes (*Vulpes lagopus*), red foxes (*Vulpes vulpes*) and hybrid foxes on fur farms in Finland [229]. An elevated mortality rate, ranging from 2 to 10 times higher than normal, was observed, accompanied by symptoms characteristic of HPAI H5N1 virus infection in mammals. These included lethargy, neurological signs, diarrhea, and rapid death. PMEs revealed systemic disease signs and lung lesions. Phylogenetic analyses indicated multiple introductions of the virus from birds to farmed fur animals, while also suggesting potential intra-species transmission, given the higher number of cases and farms involved in the outbreaks [229].

With respect to pets, infections in cats have been reported in the United States, Poland, South Korea, and France. These cats demonstrated varying degrees of clinical manifestations, from subclinical, detected by serologic surveillance, to respiratory and neurological signs, with some fatal outcomes [117,242,243]. Negative results in the dogs and cats in the same household indicated an absence of inter-mammalian transmission [243]. In 2023, HPAI H5N1 virus impacted 25 domestic cats across various regions in Poland with distinct clinical signs encompassing inappetence, lethargy, hypersalivation, fever, dyspnea, abdominal pain, and occasional urinary incontinence [232]. Rapid deterioration was anticipated by neurological manifestations such as trismus, epileptic seizures, and limb stiffness. PMEs of 11 individuals unveiled systemic inflammation accompanied by generalized congestion and swelling of internal organs. Breathing impairment was attributed to turbid fluid in the respiratory portion of the pharynx, and the lungs displayed congestion along with alveolar hemorrhages, interspersed with areas of both lighter emphysema and darker atelectasis. The cranial cavity and subdural space contained bloody fluid, with congested superficial brain vessels. Notably, the highest AIV viral loads were detected in the brain, lungs, and bronchi [232]. 

In late March and early April 2024, Texas reported the detection of HPAI H5 clade 2.3.4.4b in several cats from farms affected by the same virus infections in dairy cows. This may suggest the virus spread to the cats either from affected dairy cows, raw cow milk, or from wild birds associated with those farms [242]. Affected cats manifested neurologic signs and other clinicopathological presentations described previously.

### 5.2. Wild Mammals

#### 5.2.1. Marine Mammals

Spillover events involving HPAI H5Nx viruses of clade 2.3.4.4b to marine mammals are widely observed in seals. Specifically, along the Baltic coast of Poland during 2016–2017, an HPAI H5N8 virus was isolated from lung samples taken from two gray seals (*Halichoerus grypus*) [244]. Examination of the lungs by gross pathology and histopathology did not reveal any suspicious lesions that indicated an influenza virus infection that was diagnosed by PCR analysis [244]. Subsequent reports emerged in the UK in late 2020, involving harbor seals (*Phoca vitulina*) and a gray seal that either perished or was humanely euthanized within a 2-day span from the onset of clinical signs at a wildlife rehabilitation center [170]. Affected seals had been housed in proximity to swans, which were the first to succumb to HPAI H5N8 clade 2.3.4.4b infection at the center. Both species of seal exhibited an abrupt onset of systemic or neurological clinical signs. Harbor seals manifested seizures followed by death, while the gray seal displayed fever, facial twitching, and stupor, necessitating euthanasia on animal welfare grounds. PME revealed generalized lymphadenomegaly, occasionally coupled with multiple pale foci in the lungs, as well as congested meninges. Microscopic evaluation unveiled severe necrotizing nonsuppurative polioencephalitis associated with abundant viral antigens in neurons and ependymal cells. Surprisingly, PCR testing yielded negative results for AIV antigens in nasal and rectal swab samples of the three seals. In another occurrence, adult harbor seals were reported deceased in mid-August 2021 along the German North Sea due to HPAI H5N8 infection [245]. Histopathological assessments revealed mild to moderate lymphohistiocytic meningoencephalitis, accompanied by limited neutrophils and hemorrhage, indicating a fatal CNS infection. Further lesions encompassed moderate multifocal lymphohistiocytic vasculitis, along with single-cell necrosis, which primarily affects glial cells. Eosinophilic, shrunken, necrotic neurons, as well as fading neurons, were observed within the cerebrum and cerebellum. One individual exhibited severe, diffuse, acute lung hyperemia alongside alveolar emphysema and edema. Intriguingly, AIV antigen was not detected in the lungs, although it was present in brain tissue. Notably, tracheal swabs from the affected seals tested negative for influenza virus genomes, and lung tissues displayed notably lower viral genome loads compared to brain tissue (cycle threshold Ct value = 30–38 versus Ct = 18–22, respectively) [245]. Following the introduction of HPAI H5Nx clade 2.3.4.4b viruses into North America through migratory wild birds in 2021 [72,73], non-reassortant Eurasian 2.3.4.4b viruses were detected in harbor and gray seals stranded in Maine in June 2022, as reported by Puryear et al. [246]. Although most seals were found deceased, respiratory symptoms and, less frequently, neurological impairment were noticed prior to death. The respiratory tract was the most consistent source of AIV PCR–positive samples [246]. 

By the end of 2022, HPAI H5Nx viruses of clade 2.3.4.4b reached South America, posing a threat to both marine and terrestrial wild birds [247]. After instances of mass die-offs among wild aquatic birds, mass mortalities in sea lions (*Otaria flavescens*) have been documented along Peruvian coastlines [81,84]. Dying individuals displayed predominantly neurological clinical signs, including tremors, convulsions, and paralysis [84]. Additionally, respiratory indicators such as dyspnea, tachypnea, nasal and buccal secretions, as well as pulmonary edema, were observed. The overall good body condition of the necropsied individuals highlighted the rapid nature of death. Copious whitish secretions were filling the trachea and pharynx, alongside pulmonary congestion and hemorrhages indicative of interstitial pneumonia. Meningoencephalitis was suspected upon observation of diffuse congestion and hemorrhage [84].

Infections of cetacean species with HPAI H5Nx viruses of clade 2.3.4.4b have seldom been documented [117]. Reports include a common bottlenose dolphin (*Tursiops truncatus*) out of the coast of Florida (US) and a harbor porpoise (*Phocoena phocoena*) found stranded with overt neurological signs in shallow waters off the west coast of Sweden in 2022. In both cases, macroscopic findings were minimal, while microscopically, they presented moderate to marked lymphohistiocytic meningoencephalitis accompanied by neuronal necrosis, gliosis, perivascular cuffing, and vasculitis [248,249]. The brain exhibited the highest viral load, followed by the spinal cord, lungs, and stomach in the dolphin, and by the lungs, liver, and spleen (Ct values > 30) in the harbor porpoise [249]. IHC and in situ hybridization (ISH) confirmed widespread virus distribution within the CNS of the bottlenose dolphin [248]. In the harbor porpoise, IHC demonstrated the presence of virus antigen primarily in the brain, located in the nuclei and cytoplasm of neurons, glial cells, and epithelial cells of the choroid plexus. A scarce amount of virus antigen was detected in alveolar macrophages and sloughed epithelium of the harbor porpoise, while no viral antigen was observed in other examined tissues, consistent with findings by Murawski et al. [248]. Interestingly, sequence analysis of the HPAI H5N1 clade 2.3.4.4b virus isolates affecting these cetaceans revealed that the only genetic marker associated with mammalian adaptation was the HA-T192I (H3 numbering) amino acid substitution, linked to increased α2,6-sialic acid binding [248,250]. The overt neurotropism of this virus in marine mammals in the absence of the most common markers of mammalian adaptation underscores the potential threat of HPAI viruses to mammalian hosts [249].

#### 5.2.2. Terrestrial Mammals

Infections of HPAIV H5 clade 2.3.4.4b have been documented in a variety of wild mammalian species: squirrel, red fox, bear, large felines, bottlenose dolphin, coyote, fisher, otter, raccoon, skunk, opossum, sable, and martens. A complete list of mammalian detections can be found reported by the Animal and Plant Health Inspection Service (APHIS, U.S. Department of Agriculture) and the World Organization for Animal Health (WOAH) [251,252].

The first virological confirmation of HPAI H5 of clade 2.3.4.4b infection in mammals was in a red fox, reported in the UK in late 2020 [170]. The animal was found dead after the sudden onset of malaise and inappetence in a wildlife rehabilitation center where HPAI H5N8-infected swans were kept. Histopathological examination unveiled severe acute necrotizing nonsuppurative polioencephalitis, accompanied by mild acute nonsuppurative myocarditis and interstitial pneumonia, suggesting systemic virus replication. During the spring of 2021, additional cases of HPAI infection in red foxes emerged in the Netherlands [174]. Specifically, two cubs exhibited neurological symptoms and, after ruling out lyssavirus infection, both tested positive for HPAI H5N1 virus detection in brain samples. Genetic analysis indicated these cases might have been linked to the extensive HPAIV outbreak in barnacle geese (*Branta canadensis*) in the same region during that period [174]. Bordes et al. [253] also reported further instances of HPAI H5N1 detection in deceased red foxes in the Netherlands. While pathological examination revealed respiratory lesions due to concurrent parasitic infection in all three foxes, strong virus protein expression was observed in the brain through IHC, most notably in the cerebrum. Neurons and microglia cells in the gray matter exhibited virus protein expression, which correlated with nonsuppurative encephalitis featuring perivascular cuffing. Additionally, one individual displayed subacute lymphoplasmacytic myocarditis with myocardial degeneration and necrosis, along with virus protein expression in cardiomyocytes. Concerning the respiratory tract, virus protein expression was only identified in the olfactory epithelial cells of one fox, concomitant with necrosis of these cells. This predominant neurotropism of the virus represented a novel finding, diverging from previous HPAI H5 virus infections in foxes, which displayed a diffuse tropism with heightened virus replication in the respiratory system, akin to HPAI infections in poultry [170,254]. 

Multiple carnivore species were found to be HPAI H5N1-infected in Finland following mass mortalities of infected pheasants [186]. An otter (*Lutra lutra*), two red foxes, and a lynx (*Lynx lynx*) were examined. The observed organ lesions aligned with acute infectious diseases, featuring petechial hemorrhages in muscles, subcutaneous tissues, and multiple organs, as well as dark red, swollen, and edematous lungs. Microscopic examination of the otter revealed moderate nonsuppurative necrotizing meningoencephalitis in the cerebrum and brain stem, along with severe multifocal hemorrhagic and necrotizing pneumonia in the lungs and severe multifocal necrotizing pancreatitis. In the case of the foxes, severe multifocal necrotizing pneumonia and acute hemorrhages in the liver and kidneys were evident; however, notably, no abnormalities were observed in the brain. Lastly, the lynx exhibited multifocal acute hemorrhages in the heart and lungs, accompanied by severe necrotizing pneumonia [186].

Further cases of infection were identified among carnivores in Asia. An Ezo red fox (*Vulpes vulpes schrencki*) was found dead in a public garden in Japan in March 2022, coinciding with a crow die-off [255]. Additionally, a severely emaciated Japanese raccoon dog (*Nyctereutes procyonoides albus*) was euthanized due to its critical clinical condition [255]. Both animals tested positive for HPAI H5N1 infection, and the fox exhibited a higher virus titer in brain tissue homogenates than in the respiratory tract, as assessed by rRT-PCR (Ct = 16.29 versus Ct = 25, respectively). PME of the Ezo red fox did not reveal the presence of severe pneumonia, in contrast to previous findings in foxes [170], and no gross lesions were observed in the brain. However, localized meningoencephalitis with neutrophil infiltration and focal necrosis around the lesion were detected, albeit at a mild-to-moderate level. IHC results unveiled AIV antigen presence in the brain, bronchi, bronchioles, and trachea, indicating the virus’s neurotropism and preference for the upper respiratory tract. In the case of the raccoon dog, similar to the fox, viral antigens were detected in ciliated tracheal epithelia and tracheal glands. However, rRT-PCR analysis revealed lower viral loads in tissue homogenates from the brain, lung, and trachea. This suggested virus clearance by the immune system and implied that death resulted from parasitic infestation and secondary bacterial infections, which were identified during PME [255].

## 6. Conclusions

Since its first detection in 2013-2014, the persistent isolation of clade 2.3.4.4 in migratory waterfowl and its geographic spread are unprecedented. In poultry, HPAIV clade 2.3.4.4b infection leads to a variety of clinical outcomes, challenging early disease recognition and intervention. This clade also appears to affect a broader range of wild bird species and wild and domestic mammals, raising concerns about zoonotic events and public health implications. The severity of clinical disease, mortality, and pathological changes varies according to the host. However, both in avian and mammalian hosts, spontaneous infections were characterized by remarkable neurotropism and systemic virus spread. Given the widespread circulation of the HPAIVs clade 2.3.4.4b, further research is necessary to develop efficient strategies for preventing future outbreaks in poultry and reducing the zoonotic risk. 

## Figures and Tables

**Table 1:** Experimental infections of highly pathogenic avian influenza (HPAI) H5Nx viruses of clade 2.3.4.4b in wild waterbirds included in this review.

Species (Age)	Age	Virus	Route of Infection (Dose)	Morbidity * (%)	Mortality (%)	S or A	DT	Transmission	Ref.
Mallard	2 wk	A/Tufted-duck/Denmark/11470/LWPL/2016 (H5N8)	IC (10^3^EID_50_/mL)	0/5 (0)	0/5 (0)	n.a.	n.a.	No	[94]
			IC (10^5^EID_50_/mL)	5/5 (100)	4/5 (80)	S	5.8	Yes	
			IC (10^7^EID_50_/mL)	5/5 (100)	4/5(80)	S	3.2	Yes	
	2 wk	A/American Wigeon/South Carolina/22-000345-001/2022 (H5N1)	IC (10^3^EID_50_/mL)	5/5 (100)	1/5 (20) ^†^	S	6	Yes	[120]
			IC (10^5^EID_50_/mL)	5/5 (100)	1/5 (20) ^†^	S	4.	Yes	
			IC (10^7^EID_50_/mL)	5/5 (100)	4/5 (100) ^†^	S	5	Yes	
	6 wk	A/herring gull/Poland/MB082B/2016 (H5N8)	IO and IN (10^7^EID_50_/mL)	12/12 (100)	7/12 (58)	S	3–14	n.a.	[96]
	8 wk	A/mute swan/Shimane/ 3211A002/2017 (H5N8)	IN (10^7^EID_50_/mL)	8/8 (100)	0/8 (0)	A	n.a.	n.a.	[121]
	>9 mo	A/EurasianWigeon/NL/4/2016 (H5N8)	IC (10^7^TCID_50_/mL)	7/7 (100)	0/7 (0)	A	n.a.	n.a.	[122]
Tufted duck	>9 mo		IC (10^7^TCID_50_/mL)	7/7 (100)	1/7 (14)	S	4	n.a.	
Eurasian wigeon	7 wk	A/duck/Neth/16014829-001005/2016 (H5N8)	IO and IT (10^6^EID_50_/mL)	9/10 (90)	2/10 (20)	A	7–8	n.a.	[123]
		A/duck/Neth/17017236-001005/2017 (H5N6)	IO and IT (10^6^EID_50_/mL)	10/10 (100)	9/10 (90)	S	3–6	n.a.	
Common teal	(n.d.)	A/mute swan/Shimane/ 3211A002/2017 (H5N8)	IN (10^7^EID_50_/mL)	8/8 (100)	0/8 (0)	A	n.a.	n.a.	[124]
Herring gull	8 wk	A/herring gull/Poland/MB082B/2016 (H5N8)	IO and IN (10^7^EID_50_/mL)	12/12 (100)	11/12 (90)	S	1–6	Yes	[125]

S, symptomatic; A, asymptomatic infection; MDT, mean death time (days); IC, intrachoanal route; IN, intranasal route; IO, intraocular route; IT, intratracheal route; IV, intravenous route. n.a., not applicable. n.d., not defined. ^†^ Number of individuals showing neurological signs that were euthanized. * Infected individuals based on RT-PCR or virus isolation or hemagglutination inhibition assay.

## Data Availability

All data used are included within the manuscript.

## References

[1] Walker P.J., Siddell S.G., Lefkowitz E.J., Mushegian A.R., Adriaenssens E.M., Alfenas-Zerbini P., Dempsey D.M., Dutilh B.E., Garcia M.L., Curtis Hendrickson R. (2022). Recent changes to virus taxonomy ratified by the International Committee on Taxonomy of Viruses (2022). Arch. Virol..

[2] Schoch C.L., Ciufo S., Domrachev M., Hotton C.L., Kannan S., Khovanskaya R., Leipe D., McVeigh R., O’Neill K., Robbertse B. (2020). NCBI Taxonomy: A comprehensive update on curation, resources and tools. Database.

[3] Muramoto Y., Noda T., Kawakami E., Akkina R., Kawaoka Y. (2013). Identification of novel influenza A virus proteins translated from PA mRNA. J. Virol..

[4] Hutchinson E.C., Charles P.D., Hester S.S., Thomas B., Trudgian D., Martínez-Alonso M., Fodor E. (2014). Conserved and host-specific features of influenza virion architecture. Nat. Commun..

[5] McCrone J.T., Woods R.J., Martin E.T., Malosh R.E., Monto A.S., Lauring A.S. (2018). Stochastic processes constrain the within and between host evolution of influenza virus. eLife.

[6] Lamb R., Krug R., Knipe D.M., Howley P.M. (2001). Orthomyxoviridae: The Viruses and Their Replication. Fields Virology.

[7] Fereidouni S., Starick E., Karamendin K., Genova C.D., Scott S.D., Khan Y., Harder T., Kydyrmanov A. (2023). Genetic characterization of a new candidate hemagglutinin subtype of influenza A viruses. Emerg. Microbes Infect..

[8] Kandeil A., Gomaa M.R., Shehata M.M., El Taweel A.N., Mahmoud S.H., Bagato O., Moatasim Y., Kutkat O., Kayed A.S., Dawson P. (2019). Isolation and Characterization of a Distinct Influenza a Virus from Egyptian Bats. J. Virol..

[9] Tong S., Zhu X., Li Y., Shi M., Zhang J., Bourgeois M., Yang H., Chen X., Recuenco S., Gomez J. (2013). New world bats harbor diverse influenza A viruses. PLoS Pathog..

[10] Wu Y., Wu Y., Tefsen B., Shi Y., Gao G.F. (2014). Bat-derived influenza-like viruses H17N10 and H18N11. Trends Microbiol..

[11] Alexander D.J. (2007). An overview of the epidemiology of avian influenza. Vaccine.

[12] Alexander D.J. (2000). A review of avian influenza in different bird species. Vet. Microbiol..

[13] Swayne D.E., Suarez D.L. (2000). Highly pathogenic avian influenza. Rev. Sci. Tech..

[14] World Organization for Animal Health International Animal Health Code. https://www.woah.org/en/what-we-do/standards/codes-and-manuals/terrestrial-code-online-access/.

[15] World Organisation for Animal Health Infection with High Pathogenicity Avian Influenza Viruses. https://www.woah.org/fileadmin/Home/eng/Health_standards/tahc/2023/chapitre_avian_influenza_viruses.pdf.

[16] Xu X., Subbarao K., Cox N.J., Guo Y. (1999). Genetic characterization of the pathogenic influenza A/Goose/Guangdong/1/96 (H5N1) virus: Similarity of its hemagglutinin gene to those of H5N1 viruses from the 1997 outbreaks in Hong Kong. Virology.

[17] Guo Y., Xu X., Wan X. (1998). Genetic characterization of an avian influenza A (H5N1) virus isolated from a sick goose in China. Zhonghua Shi Yan He Lin Chuang Bing Du Xue Za Zhi.

[18] Marandino A., Tomás G., Panzera Y., Leizagoyen C., Pérez R., Bassetti L., Negro R., Rodríguez S., Pérez R. (2023). Spreading of the High-Pathogenicity Avian Influenza (H5N1) Virus of Clade 2.3.4.4b into Uruguay. Viruses.

[19] Lee D.H., Bertran K., Kwon J.H., Swayne D.E. (2017). Evolution, global spread, and pathogenicity of highly pathogenic avian influenza H5Nx clade 2.3.4.4. J. Vet. Sci..

[20] Kandeil A., Patton C., Jones J.C., Jeevan T., Harrington W.N., Trifkovic S., Seiler J.P., Fabrizio T., Woodard K., Turner J.C. (2023). Rapid evolution of A(H5N1) influenza viruses after intercontinental spread to North America. Nat. Commun..

[21] Lee D.H., Criado M.F., Swayne D.E. (2021). Pathobiological Origins and Evolutionary History of Highly Pathogenic Avian Influenza Viruses. Cold Spring Harb. Perspect. Med..

[22] Pantin-Jackwood M.J., Swayne D.E. (2007). Pathobiology of Asian highly pathogenic avian influenza H5N1 virus infections in ducks. Avian Dis..

[23] United States Department of Agriculture, Animal and Plant Health Inspection Service USDA, FDA and CDC Share Update on HPAI Detections in Dairy Cattle. https://www.aphis.usda.gov/news/agency-announcements/usda-fda-cdc-share-update-hpai-detections-dairy-cattle.

[24] Thompson A.J., Paulson J.C. (2021). Adaptation of influenza viruses to human airway receptors. J. Biol. Chem..

[25] Swayne D.E. (2007). Understanding the complex pathobiology of high pathogenicity avian influenza viruses in birds. Avian Dis..

[26] Long J.S., Mistry B., Haslam S.M., Barclay W.S. (2019). Host and viral determinants of influenza A virus species specificity. Nat. Rev. Microbiol..

[27] Steinhauer D.A. (1999). Role of hemagglutinin cleavage for the pathogenicity of influenza virus. Virology.

[28] Wiley D.C., Skehel J.J. (1987). The structure and function of the hemagglutinin membrane glycoprotein of influenza virus. Annu. Rev. Biochem..

[29] Wilson I.A., Skehel J.J., Wiley D.C. (1981). Structure of the haemagglutinin membrane glycoprotein of influenza virus at 3 Å resolution. Nature.

[30] Swayne D.E. (2009). Avian Influenza.

[31] Chen J., Lee K.H., Steinhauer D.A., Stevens D.J., Skehel J.J., Wiley D.C. (1998). Structure of the hemagglutinin precursor cleavage site, a determinant of influenza pathogenicity and the origin of the labile conformation. Cell.

[32] Gotoh B., Ogasawara T., Toyoda T., Inocencio N.M., Hamaguchi M., Nagai Y. (1990). An endoprotease homologous to the blood clotting factor X as a determinant of viral tropism in chick embryo. EMBO J..

[33] Klenk H.D., Garten W. (1994). Host cell proteases controlling virus pathogenicity. Trends Microbiol..

[34] Stieneke-Gröber A., Vey M., Angliker H., Shaw E., Thomas G., Roberts C., Klenk H.D., Garten W. (1992). Influenza virus hemagglutinin with multibasic cleavage site is activated by furin, a subtilisin-like endoprotease. EMBO J..

[35] Swayne D.E., Pantin-Jackwood M. (2006). Pathogenicity of avian influenza viruses in poultry. Dev. Biol..

[36] Hulse D.J., Webster R.G., Russell R.J., Perez D.R. (2004). Molecular determinants within the surface proteins involved in the pathogenicity of H5N1 influenza viruses in chickens. J. Virol..

[37] World Organisation for Animal Health (2012). Avian Influenza. Manual of Diagnostic Tests and Vaccines for Terrestrial Animals.

[38] Wilson I.A., Cox N.J. (1990). Structural basis of immune recognition of influenza virus hemagglutinin. Annu. Rev. Immunol..

[39] Marcelin G., Sandbulte M.R., Webby R.J. (2012). Contribution of antibody production against neuraminidase to the protection afforded by influenza vaccines. Rev. Med. Virol..

[40] Johansson B.E., Matthews J.T., Kilbourne E.D. (1998). Supplementation of conventional influenza A vaccine with purified viral neuraminidase results in a balanced and broadened immune response. Vaccine.

[41] Suarez D.L., Schultz-Cherry S. (2000). Immunology of avian influenza virus: A review. Dev. Comp. Immunol..

[42] Cattoli G., Milani A., Temperton N., Zecchin B., Buratin A., Molesti E., Aly M.M., Arafa A., Capua I. (2011). Antigenic drift in H5N1 avian influenza virus in poultry is driven by mutations in major antigenic sites of the hemagglutinin molecule analogous to those for human influenza virus. J. Virol..

[43] Plotkin J.B., Dushoff J. (2003). Codon bias and frequency-dependent selection on the hemagglutinin epitopes of influenza A virus. Proc. Natl. Acad. Sci. USA.

[44] Bouvier N.M., Palese P. (2008). The biology of influenza viruses. Vaccine.

[45] Suttie A., Deng Y.M., Greenhill A.R., Dussart P., Horwood P.F., Karlsson E.A. (2019). Inventory of molecular markers affecting biological characteristics of avian influenza A viruses. Virus Genes.

[46] Carnaccini S., Perez D.R. (2020). H9 Influenza Viruses: An Emerging Challenge. Cold Spring Harb. Perspect. Med..

[47] Pappas C., Aguilar P.V., Basler C.F., Solórzano A., Zeng H., Perrone L.A., Palese P., García-Sastre A., Katz J.M., Tumpey T.M. (2008). Single gene reassortants identify a critical role for PB1, HA, and NA in the high virulence of the 1918 pandemic influenza virus. Proc. Natl. Acad. Sci. USA.

[48] Webster R.G., Govorkova E.A. (2014). Continuing challenges in influenza. Ann. N. Y. Acad. Sci..

[49] Smith G.J., Donis R.O. (2015). Nomenclature updates resulting from the evolution of avian influenza A(H5) virus clades 2.1.3.2a, 2.2.1, and 2.3.4 during 2013ߝ2014. Influenza Other Respir. Viruses.

[50] World Health Organization (2020). Antigenic and Genetic Characteristics of Zoonotic Influenza A Viruses and Development of Candidate Vaccine Viruses for Pandemic Preparedness.

[51] Kuiken T., Fouchier R.A.M., Koopmans M.P.G. (2023). Being ready for the next influenza pandemic?. Lancet Infect. Dis..

[52] Zhao K., Gu M., Zhong L., Duan Z., Zhang Y., Zhu Y., Zhao G., Zhao M., Chen Z., Hu S. (2013). Characterization of three H5N5 and one H5N8 highly pathogenic avian influenza viruses in China. Vet. Microbiol..

[53] Lee Y.J., Kang H.M., Lee E.K., Song B.M., Jeong J., Kwon Y.K., Kim H.R., Lee K.J., Hong M.S., Jang I. (2014). Novel reassortant influenza A(H5N8) viruses, South Korea, 2014. Emerg. Infect. Dis..

[54] Adlhoch C., Fusaro A., Gonzales J.L., Kuiken T., Marangon S., Niqueux E., Staubach C., European Food Safety Authority, European Centre for Disease Prevention and Control, European Union Reference Laboratory for Avian Influenza (2023). Avian influenza overview September—December 2022. EFSA J..

[55] Lee D.H., Torchetti M.K., Winker K., Ip H.S., Song C.S., Swayne D.E. (2015). Intercontinental Spread of Asian-Origin H5N8 to North America through Beringia by Migratory Birds. J. Virol..

[56] Lee D.H., Bahl J., Torchetti M.K., Killian M.L., Ip H.S., DeLiberto T.J., Swayne D.E. (2016). Highly Pathogenic Avian Influenza Viruses and Generation of Novel Reassortants, United States, 2014–2015. Emerg. Infect. Dis..

[57] Lee D.H., Torchetti M.K., Hicks J., Killian M.L., Bahl J., Pantin-Jackwood M., Swayne D.E. (2018). Transmission Dynamics of Highly Pathogenic Avian Influenza Virus A(H5Nx) Clade 2.3.4.4, North America, 2014–2015. Emerg. Infect. Dis..

[58] United States Department of Agriculture, Animal and Plant Health Inspection Service July 2016–June 2017 Wild Bird Highly Pathogenic Avian Influenza Cases in the United States. https://www.aphis.usda.gov/animal_health/downloads/animal_diseases/ai/uspositivecases17.pdf.

[59] Centers for Disease Control and Prevention, National Center for Immunization and Respiratory Diseases (NCIRD) Technical Report: Highly Pathogenic Avian Influenza A(H5N1) Viruses. https://www.cdc.gov/flu/avianflu/spotlights/2023-2024/h5n1-technical-report_april-2024.htm.

[60] Wu H., Peng X., Xu L., Jin C., Cheng L., Lu X., Xie T., Yao H., Wu N. (2014). Novel reassortant influenza A(H5N8) viruses in domestic ducks, eastern China. Emerg. Infect. Dis..

[61] Lee D.H., Sharshov K., Swayne D.E., Kurskaya O., Sobolev I., Kabilov M., Alekseev A., Irza V., Shestopalov A. (2017). Novel Reassortant Clade 2.3.4.4 Avian Influenza A(H5N8) Virus in Wild Aquatic Birds, Russia, 2016. Emerg. Infect. Dis..

[62] Li M., Liu H., Bi Y., Sun J., Wong G., Liu D., Li L., Liu J., Chen Q., Wang H. (2017). Highly Pathogenic Avian Influenza A(H5N8) Virus in Wild Migratory Birds, Qinghai Lake, China. Emerg. Infect. Dis..

[63] Brown I., Kuiken T., Mulatti P., Smietanka K., Staubach C., Stroud D., Therkildsen O.R., European Food Safety Authority, European Centre for Disease Prevention and Control, European Union Reference Laboratory for Avian influenza (2017). Avian influenza overview September–November 2017. EFSA J..

[64] Lycett S.J., Pohlmann A., Staubach C., Caliendo V., Woolhouse M., Beer M., Kuiken T. (2020). Genesis and spread of multiple reassortants during the 2016/2017 H5 avian influenza epidemic in Eurasia. Proc. Natl. Acad. Sci. USA.

[65] Adlhoch C., Brouwer A., Kuiken T., Mulatti P., Smietanka K., Staubach C., Willeberg P., European Food Safety Authority, European Centre for Disease Prevention and Control, European Union Reference Laboratory for Avian Influenza (2018). Avian influenza overview November 2017–February 2018. EFSA J..

[66] Adlhoch C., Brouwer A., Kuiken T., Miteva A., Mulatti P., Smietanka K., Staubach C., European Food Safety Authority, European Centre for Disease Prevention and Control, European Union Reference Laboratory for Avian Influenza (2018). Avian influenza overview August–November 2018. EFSA J..

[67] Adlhoch C., Fusaro A., Kuiken T., Niqueux E., Staubach C., Terregino C., Guajardo I.M., European Food Safety Authority, European Centre for Disease Prevention and Control, European Union Reference Laboratory for Avian Influenza (2020). Avian influenza overview November 2019–February 2020. EFSA J..

[68] Adlhoch C., Fusaro A., Gonzales J.L., Kuiken T., Marangon S., Niqueux É., Staubach C., European Food Safety Authority, European Centre for Disease Prevention Control, European Union Reference Laboratory for Avian Influenza (2021). Avian influenza overview September–December 2021. EFSA J..

[69] Adlhoch C., Fusaro A., Gonzales J.L., Kuiken T., Marangon S., Niqueux É., Staubach C., European Food Safety Authority, European Centre for Disease Prevention and Control, European Union Reference Laboratory for Avian Influenza (2021). Avian influenza overview December 2020–February 2021. EFSA J..

[70] Adlhoch C., Fusaro A., Gonzales J.L., Kuiken T., Marangon S., Niqueux E., Staubach C., European Food Safety Authority, European Centre for Disease Prevention and Control, European Union Reference Laboratory for Avian Influenza (2022). Avian influenza overview December 2021–March 2022. EFSA J..

[71] Adlhoch C., Fusaro A., Gonzales J.L., Kuiken T., Mirinavičiūtė G., Niqueux É., Staubach C., European Food Safety Authority, European Centre for Disease Prevention and Control, European Union Reference Laboratory for Avian Influenza (2023). Avian influenza overview June–September 2023. EFSA J..

[72] Bevins S.N., Shriner S.A., Cumbee J.C., Dilione K.E., Douglass K.E., Ellis J.W., Killian M.L., Torchetti M.K., Lenoch J.B. (2022). Intercontinental Movement of Highly Pathogenic Avian Influenza A(H5N1) Clade 2.3.4.4 Virus to the United States, 2021. Emerg. Infect. Dis..

[73] Caliendo V., Lewis N.S., Pohlmann A., Baillie S.R., Banyard A.C., Beer M., Brown I.H., Fouchier R.A.M., Hansen R.D.E., Lameris T.K. (2022). Transatlantic spread of highly pathogenic avian influenza H5N1 by wild birds from Europe to North America in 2021. Sci. Rep..

[74] Günther A., Krone O., Svansson V., Pohlmann A., King J., Hallgrimsson G.T., Skarphéðinsson K.H., Sigurðardóttir H., Jónsson S.R., Beer M. (2022). Iceland as Stepping Stone for Spread of Highly Pathogenic Avian Influenza Virus between Europe and North America. Emerg. Infect. Dis..

[75] Alkie T.N., Lopes S., Hisanaga T., Xu W., Suderman M., Koziuk J., Fisher M., Redford T., Lung O., Joseph T. (2022). A threat from both sides: Multiple introductions of genetically distinct H5 HPAI viruses into Canada via both East Asia-Australasia/Pacific and Atlantic flyways. Virus Evol..

[76] Youk S., Torchetti M.K., Lantz K., Lenoch J.B., Killian M.L., Leyson C., Bevins S.N., Dilione K., Ip H.S., Stallknecht D.E. (2023). H5N1 highly pathogenic avian influenza clade 2.3.4.4b in wild and domestic birds: Introductions into the United States and reassortments, December 2021–April 2022. Virology.

[77] Ramey A.M., Scott L.C., Ahlstrom C.A., Buck E.J., Williams A.R., Kim Torchetti M., Stallknecht D.E., Poulson R.L. (2024). Molecular detection and characterization of highly pathogenic H5N1 clade 2.3.4.4b avian influenza viruses among hunter-harvested wild birds provides evidence for three independent introductions into Alaska. Virology.

[78] Animal and Plant Health Inspection Service, U.S. Department of Agriculture 2022–2024 Confirmations of Highly Pathogenic Avian Influenza in Commercial and Backyard Flocks. https://www.aphis.usda.gov/aphis/ourfocus/animalhealth/animal-disease-information/avian/avian-influenza/hpai-2022/2022-hpai-commercial-backyard-flocks.

[79] Animal and Plant Health Inspection Service, U.S. Department of Agriculture (2024). 2022–2024 Detections of Highly Pathogenic Avian Influenza in Wild Birds. https://www.aphis.usda.gov/livestock-poultry-disease/avian/avian-influenza/hpai-detections/wild-birds.

[80] Adlhoch C., Fusaro A., Gonzales J.L., Kuiken T., Marangon S., Mirinaviciute G., Niqueux E., European Food Safety Authority, European Centre for Disease Prevention and Control, European Union Reference Laboratory for Avian Influenza (2023). Avian influenza overview December 2022–March 2023. EFSA J..

[81] Mariana L., Alejandra G.-G., Breno M.-S., Diana J., Patricia B., Carlos C.-M., Javier J., Walter S., Karl P., Lady A. (2023). Highly pathogenic avian influenza A (H5N1) in marine mammals and seabirds in Peru. Nat. Commun..

[82] Castro-Sanguinetti G.R., González-Veliz R., Callupe-Leyva A., Apaza-Chiara A.P., Jara J., Silva W., Icochea E., More-Bayona J.A. (2024). Highly pathogenic avian influenza virus H5N1 clade 2.3.4.4b from Peru forms a monophyletic group with Chilean isolates in South America. Sci. Rep..

[83] Gamarra-Toledo V., Plaza P.I., Angulo F., Gutiérrez R., García-Tello O., Saravia-Guevara P., Mejía-Vargas F., Epiquién-Rivera M., Quiroz-Jiménez G., Martinez P. (2023). Highly Pathogenic Avian Influenza (HPAI) strongly impacts wild birds in Peru. Biol. Conserv..

[84] Gamarra-Toledo V., Plaza P.I., Gutiérrez R., Inga-Diaz G., Saravia-Guevara P., Pereyra-Meza O., Coronado-Flores E., Calderón-Cerrón A., Quiroz-Jiménez G., Martinez P. (2023). Mass Mortality of Marine Mammals Associated to Highly Pathogenic Influenza Virus (H5N1) in South America. bioRxiv.

[85] Reischak D., Rivetti A.V., Otaka J.N.P., Domingues C.S., Freitas T.d.L., Cardoso F.G., Montesino L.O., da Silva A.L.S., Malta F., Amgarten D. (2023). First report and genetic characterization of the highly pathogenic avian influenza A(H5N1) virus in Cabot’s tern (*Thalasseus acuflavidus*), Brazil. Vet. Anim. Sci..

[86] Rimondi A., Vanstreels R.E.T., Olivera V., Donini A., Lauriente M.M., Uhart M. (2024). Highly Pathogenic Avian Influenza A(H5N1) Viruses from Multispecies Outbreak, Argentina, August 2023. Emerg. Infect. Dis..

[87] Bennison A., Byrne A.M.P., Reid S.M., Lynton-Jenkins J.G., Mollett B., Sliva D.D., Peers-Dent J., Finlayson K., Hall R., Blockley F. (2023). Detection and spread of high pathogenicity avian influenza virus H5N1 in the Antarctic Region. bioRxiv.

[88] Adlhoch C., Fusaro A., Gonzales J.L., Kuiken T., Mirinavičiūtė G., Niqueux É., Ståhl K., European Food Safety Authority, European Centre for Disease Prevention and Control, European Union Reference Laboratory for Avian Influenza (2023). Avian influenza overview September–December 2023. EFSA J..

[89] Bi Y., Chen Q., Wang Q., Chen J., Jin T., Wong G., Quan C., Liu J., Wu J., Yin R. (2016). Genesis, Evolution and Prevalence of H5N6 Avian Influenza Viruses in China. Cell Host Microbe.

[90] Kwon J.H., Noh Y.K., Lee D.H., Yuk S.S., Erdene-Ochir T.O., Noh J.Y., Hong W.T., Jeong J.H., Jeong S., Gwon G.B. (2017). Experimental infection with highly pathogenic H5N8 avian influenza viruses in the Mandarin duck (*Aix galericulata*) and domestic pigeon (*Columba livia domestica*). Vet. Microbiol..

[91] Takemae N., Tsunekuni R., Sharshov K., Tanikawa T., Uchida Y., Ito H., Soda K., Usui T., Sobolev I., Shestopalov A. (2017). Five distinct reassortants of H5N6 highly pathogenic avian influenza A viruses affected Japan during the winter of 2016–2017. Virology.

[92] Elbe S., Buckland-Merrett G. (2017). Data, disease and diplomacy: GISAID’s innovative contribution to global health. Glob. Chall..

[93] Koethe S., Ulrich L., Ulrich R., Amler S., Graaf A., Harder T.C., Grund C., Mettenleiter T.C., Conraths F.J., Beer M. (2020). Modulation of lethal HPAIV H5N8 clade 2.3.4.4B infection in AIV pre-exposed mallards. Emerg. Microbes Infect..

[94] Leyson C., Youk S.S., Smith D., Dimitrov K., Lee D.H., Larsen L.E., Swayne D.E., Pantin-Jackwood M.J. (2019). Pathogenicity and genomic changes of a 2016 European H5N8 highly pathogenic avian influenza virus (clade 2.3.4.4) in experimentally infected mallards and chickens. Virology.

[95] Kim Y.I., Pascua P.N., Kwon H.I., Lim G.J., Kim E.H., Yoon S.W., Park S.J., Kim S.M., Choi E.J., Si Y.J. (2014). Pathobiological features of a novel, highly pathogenic avian influenza A(H5N8) virus. Emerg. Microbes Infect..

[96] Tarasiuk K., Kycko A., Świętoń E., Bocian Ł., Wyrostek K., Śmietanka K. (2023). Homo- and Heterosubtypic Immunity to Low Pathogenic Avian Influenza Virus Mitigates the Clinical Outcome of Infection with Highly Pathogenic Avian Influenza H5N8 Clade 2.3.4.4.b in Captive Mallards (*Anas platyrhynchos*). Pathogens.

[97] Gobbo F., Fornasiero D., De Marco M.A., Zecchin B., Mulatti P., Delogu M., Terregino C. (2021). Active Surveillance for Highly Pathogenic Avian Influenza Viruses in Wintering Waterbirds in Northeast Italy, 2020–2021. Microorganisms.

[98] Kleyheeg E., Slaterus R., Bodewes R., Rijks J.M., Spierenburg M.A.H., Beerens N., Kelder L., Poen M.J., Stegeman J.A., Fouchier R.A.M. (2017). Deaths among Wild Birds during Highly Pathogenic Avian Influenza A(H5N8) Virus Outbreak, the Netherlands. Emerg. Infect. Dis..

[99] Aznar I., Baldinelli F., Papanikolaou A., Stoicescu A., Van der Stede Y., European Food Safety Authority (2021). Annual Report on surveillance for avian influenza in poultry and wild birds in Member States of the European Union in 2020. EFSA J..

[100] Aznar I., Baldinelli F., Stoicescu A., Kohnle L., European Food Safety Authority (2022). Annual report on surveillance for avian influenza in poultry and wild birds in Member States of the European Union in 2021. EFSA J..

[101] Pohlmann A., Stejskal O., King J., Bouwhuis S., Packmor F., Ballstaedt E., Hälterlein B., Hennig V., Stacker L., Graaf A. (2023). Mass mortality among colony-breeding seabirds in the German Wadden Sea in 2022 due to distinct genotypes of HPAIV H5N1 clade 2.3.4.4b. J. Gen. Virol..

[102] Bodewes R., Kuiken T. (2018). Changing Role of Wild Birds in the Epidemiology of Avian Influenza A Viruses. Adv. Virus Res..

[103] Pohlmann A., King J., Fusaro A., Zecchin B., Banyard A.C., Brown I.H., Byrne A.M.P., Beerens N., Liang Y., Heutink R. (2022). Has Epizootic Become Enzootic? Evidence for a Fundamental Change in the Infection Dynamics of Highly Pathogenic Avian Influenza in Europe, 2021. mBio.

[104] CMS FAO CO-Convened Scientific Task Force on Avian Influenza and Wild Birds Scientific Task Force on Avian Influenza and Wild Birds Statement–July 2023. https://www.cms.int/sites/default/files/publication/avian_influenza_2023_aug.pdf.

[105] IUCN The IUCN Red List of Threatened Species. Version 2022-2. https://www.iucnredlist.org.

[106] EAAFP Updates of HPAI recorded in East Asian–Australasian Flyway. https://www.eaaflyway.net/updates-hpai-eaaf/.

[107] Alexandrou O., Malakou M., Catsadorakis G. (2022). The impact of avian influenza 2022 on Dalmatian pelicans was the worst ever wildlife disaster in Greece. Oryx.

[108] Cunningham E.J.A., Gamble A., Hart T., Humphreys E.M., Philip E., Tyler G., Wood M.J. (2022). The incursion of Highly Pathogenic Avian Influenza (HPAI) into North Atlantic seabird populations: An interim report from the 15th International Seabird Group conference. Seabird.

[109] U.S. Fish & Wildlife Service California Condors & HPAI Update. https://www.fws.gov/program/california-condor-recovery/southwest-california-condor-flock-hpai-information-updates-2023.

[110] Animal and Plant Health Inspection Service, U.S. Department of Agriculture USDA Takes Action to Help Protect Endangered California Condors from Highly Pathogenic Avian Influenza. https://usbiotechnologyregulation.mrp.usda.gov/aphis/newsroom/stakeholder-info/sa_by_date/sa-2023/ca-condor-hpai.

[111] Stokstad E. (2023). Deadly avian flu reaches Galápagos Islands. Science.

[112] Gobbo F., Zanardello C., Bottinelli M., Budai J., Bruno F., De Nardi R., Patregnani T., Catania S., Terregino C. (2022). Silent Infection of Highly Pathogenic Avian Influenza Virus (H5N1) Clade 2.3.4.4b in a Commercial Chicken Broiler Flock in Italy. Viruses.

[113] Song B.M., Kang H.M., Lee E.K., Jung J., Kang Y., Lee H.S., Lee Y.J. (2015). Pathogenicity of H5N8 virus in chickens from Korea in 2014. J. Vet. Sci..

[114] Takadate Y., Tsunekuni R., Kumagai A., Mine J., Kikutani Y., Sakuma S., Miyazawa K., Uchida Y. (2023). Different Infectivity and Transmissibility of H5N8 and H5N1 High Pathogenicity Avian Influenza Viruses Isolated from Chickens in Japan in the 2021/2022 Season. Viruses.

[115] Kwon J.H., Bertran K., Lee D.H., Criado M.F., Killmaster L., Pantin-Jackwood M.J., Swayne D.E. (2023). Diverse infectivity, transmissibility, and pathobiology of clade 2.3.4.4 H5Nx highly pathogenic avian influenza viruses in chickens. Emerg. Microbes Infect..

[116] Adlhoch C., Fusaro A., Gonzales J.L., Kuiken T., Marangon S., Niqueux E., Staubach C., European Food Safety Authority, European Centre for Disease Prevention and Control, European Union Reference Laboratory for Avian Influenza (2022). Avian influenza overview March–June 2022. EFSA J..

[117] Adlhoch C., Fusaro A., Gonzales J.L., Kuiken T., Melidou A., Mirinavičiūtė G., Niqueux É., European Food Safety Authority, European Centre for Disease Prevention and Control, European Union Reference Laboratory for Avian Influenza (2023). Avian influenza overview April–June 2023. EFSA J..

[118] Vreman S., Kik M., Germeraad E., Heutink R., Harders F., Spierenburg M., Engelsma M., Rijks J., van den Brand J., Beerens N. (2023). Zoonotic Mutation of Highly Pathogenic Avian Influenza H5N1 Virus Identified in the Brain of Multiple Wild Carnivore Species. Pathogens.

[119] Appleby M.C., Mench J.A., Hughes B.O. (2004). Poultry Behaviour and Welfare.

[120] Spackman E., Pantin-Jackwood M.J., Lee S.A., Prosser D. (2023). The pathogenesis of a 2022 North American highly pathogenic clade 2.3.4.4b H5N1 avian influenza virus in mallards (*Anas platyrhynchos*). Avian Pathol..

[121] Tanikawa T., Fujii K., Sugie Y., Tsunekuni R., Nakayama M., Kobayashi S. (2022). Comparative susceptibility of mallard (*Anas platyrhynchos*) to infection with high pathogenicity avian influenza virus strains (Gs/Gd lineage) isolated in Japan in 2004–2017. Vet. Microbiol..

[122] Caliendo V., Leijten L., van de Bildt M.W.G., Poen M.J., Kok A., Bestebroer T., Richard M., Fouchier R.A.M., Kuiken T. (2022). Long-Term Protective Effect of Serial Infections with H5N8 Highly Pathogenic Avian Influenza Virus in Wild Ducks. J. Virol..

[123] Beerens N., Germeraad E.A., Venema S., Verheij E., Pritz-Verschuren S.B.E., Gonzales J.L. (2021). Comparative pathogenicity and environmental transmission of recent highly pathogenic avian influenza H5 viruses. Emerg. Microbes Infect..

[124] Tanikawa T., Sakuma S., Yoshida E., Tsunekuni R., Nakayama M., Kobayashi S. (2021). Comparative susceptibility of the common teal (*Anas crecca*) to infection with high pathogenic avian influenza virus strains isolated in Japan in 2004–2017. Vet. Microbiol..

[125] Tarasiuk K., Kycko A., Knitter M., Świętoń E., Wyrostek K., Domańska-Blicharz K., Bocian Ł., Meissner W., Śmietanka K. (2022). Pathogenicity of highly pathogenic avian influenza H5N8 subtype for herring gulls (*Larus argentatus*): Impact of homo- and heterosubtypic immunity on the outcome of infection. Vet. Res..

[126] Kim J.K., Negovetich N.J., Forrest H.L., Webster R.G. (2009). Ducks: The “Trojan horses” of H5N1 influenza. Influenza Other Respir. Viruses.

[127] Campbell L.K., Magor K.E. (2020). Pattern Recognition Receptor Signaling and Innate Responses to Influenza A Viruses in the Mallard Duck, Compared to Humans and Chickens. Front. Cell. Infect. Microbiol..

[128] Webster R.G., Bean W.J., Gorman O.T., Chambers T.M., Kawaoka Y. (1992). Evolution and ecology of influenza A viruses. Microbiol. Rev..

[129] Evseev D., Magor K.E. (2019). Innate Immune Responses to Avian Influenza Viruses in Ducks and Chickens. Vet. Sci..

[130] Barber M.R.W., Aldridge J.R., Webster R.G., Magor K.E. (2010). Association of RIG-I with innate immunity of ducks to influenza. Proc. Natl. Acad. Sci. USA.

[131] Grund C., Hoffmann D., Ulrich R., Naguib M., Schinköthe J., Hoffmann B., Harder T., Saenger S., Zscheppang K., Tönnies M. (2018). A novel European H5N8 influenza A virus has increased virulence in ducks but low zoonotic potential. Emerg. Microbes Infect..

[132] Kang H.M., Choi J.G., Kim K.I., Kim B.S., Batchuluun D., Erdene-Ochir T.O., Kim M.C., Kwon J.H., Park C.K., Lee Y.J. (2013). Pathogenicity in domestic ducks and mice of highly pathogenic H5N1 clade 2.3.2.1 influenza viruses recently circulating in Eastern Asia. Vet. Microbiol..

[133] Uchida Y., Mine J., Takemae N., Tanikawa T., Tsunekuni R., Saito T. (2019). Comparative pathogenicity of H5N6 subtype highly pathogenic avian influenza viruses in chicken, Pekin duck and Muscovy duck. Transbound. Emerg. Dis..

[134] Leyson C.M., Youk S., Ferreira H.L., Suarez D.L., Pantin-Jackwood M. (2021). Multiple Gene Segments Are Associated with Enhanced Virulence of Clade 2.3.4.4 H5N8 Highly Pathogenic Avian Influenza Virus in Mallards. J. Virol..

[135] Slomka M.J., Puranik A., Mahmood S., Thomas S.S., Seekings A.H., Byrne A.M.P., Núñez A., Bianco C., Mollett B.C., Watson S. (2019). Ducks Are Susceptible to Infection with a Range of Doses of H5N8 Highly Pathogenic Avian Influenza Virus (2016, Clade 2.3.4.4b) and Are Largely Resistant to Virus-Specific Mortality, but Efficiently Transmit Infection to Contact Turkeys. Avian Dis..

[136] Lee I.H., Jin S.Y., Seo S.H. (2017). Genetic and pathogenic analysis of a novel reassortant H5N6 influenza virus isolated from waterfowl in South Korea in 2016. Arch. Virol..

[137] Pantin-Jackwood M.J., Costa-Hurtado M., Shepherd E., DeJesus E., Smith D., Spackman E., Kapczynski D.R., Suarez D.L., Stallknecht D.E., Swayne D.E. (2016). Pathogenicity and Transmission of H5 and H7 Highly Pathogenic Avian Influenza Viruses in Mallards. J. Virol..

[138] Berhane Y., Kobasa D., Embury-Hyatt C., Pickering B., Babiuk S., Joseph T., Bowes V., Suderman M., Leung A., Cottam-Birt C. (2016). Pathobiological Characterization of a Novel Reassortant Highly Pathogenic H5N1 Virus Isolated in British Columbia, Canada, 2015. Sci. Rep..

[139] Pantin-Jackwood M.J., Costa-Hurtado M., Bertran K., DeJesus E., Smith D., Swayne D.E. (2017). Infectivity, transmission and pathogenicity of H5 highly pathogenic avian influenza clade 2.3.4.4 (H5N8 and H5N2) United States index viruses in Pekin ducks and Chinese geese. Vet. Res..

[140] Hinshaw V.S., Webster R.G., Turner B. (1980). The perpetuation of orthomyxoviruses and paramyxoviruses in Canadian waterfowl. Can. J. Microbiol..

[141] Webster R.G., Yakhno M., Hinshaw V.S., Bean W.J., Copal Murti K. (1978). Intestinal influenza: Replication and characterization of influenza viruses in ducks. Virology.

[142] Caliendo V., Leijten L., Begeman L., Poen M.J., Fouchier R.A.M., Beerens N., Kuiken T. (2020). Enterotropism of highly pathogenic avian influenza virus H5N8 from the 2016/2017 epidemic in some wild bird species. Vet. Res..

[143] Caliendo V., Leijten L., van de Bildt M., Germeraad E., Fouchier R.A.M., Beerens N., Kuiken T. (2022). Tropism of Highly Pathogenic Avian Influenza H5 Viruses from the 2020/2021 Epizootic in Wild Ducks and Geese. Viruses.

[144] Lean F.Z.X., Vitores A.G., Reid S.M., Banyard A.C., Brown I.H., Núñez A., Hansen R.D.E. (2022). Gross pathology of high pathogenicity avian influenza virus H5N1 2021–2022 epizootic in naturally infected birds in the United Kingdom. One Health.

[145] Lean F.Z.X., Núñez A., Banyard A.C., Reid S.M., Brown I.H., Hansen R.D.E. (2022). Gross pathology associated with highly pathogenic avian influenza H5N8 and H5N1 in naturally infected birds in the UK (2020–2021). Vet. Rec..

[146] Adlhoch C., Fusaro A., Gonzales J.L., Kuiken T., Marangon S., Niqueux É., Staubach C., European Food Safety Authority, European Centre for Disease Prevention and Control, European Union Reference Laboratory for Avian Influenza (2021). Avian influenza overview February–May 2021. EFSA J..

[147] Animal and Plant Health Agency Annual Report on Surveillance for Avian Influenza in Poultry and Wild Birds in Member States of the European Union in 2017. https://food.ec.europa.eu/system/files/2018-11/ad_control-measures_ai_surv-rslt_pltry-wld-brds_2017.pdf.

[148] Brown J.D., Stallknecht D.E., Swayne D.E. (2008). Experimental infection of swans and geese with highly pathogenic avian influenza virus (H5N1) of Asian lineage. Emerg. Infect. Dis..

[149] Fereidouni S.R., Starick E., Beer M., Wilking H., Kalthoff D., Grund C., Häuslaigner R., Breithaupt A., Lange E., Harder T.C. (2009). Highly Pathogenic Avian Influenza Virus Infection of Mallards with Homo- and Heterosubtypic Immunity Induced by Low Pathogenic Avian Influenza Viruses. PLoS ONE.

[150] Costa T.P., Brown J.D., Howerth E.W., Stallknecht D.E., Swayne D.E. (2011). Homo- and heterosubtypic low pathogenic avian influenza exposure on H5N1 highly pathogenic avian influenza virus infection in wood ducks (*Aix sponsa*). PLoS ONE.

[151] Lv X., Li X., Sun H., Li Y., Peng P., Qin S., Wang W., Li Y., An Q., Fu T. (2022). Highly Pathogenic Avian Influenza A(H5N8) Clade 2.3.4.4b Viruses in Satellite-Tracked Wild Ducks, Ningxia, China, 2020. Emerg. Infect. Dis..

[152] Verhagen J.H., van der Jeugd H.P., Nolet B.A., Slaterus R., Kharitonov S.P., de Vries P.P., Vuong O., Majoor F., Kuiken T., Fouchier R.A. (2015). Wild bird surveillance around outbreaks of highly pathogenic avian influenza A(H5N8) virus in the Netherlands, 2014, within the context of global flyways. Euro Surveill..

[153] Poen M.J., Verhagen J.H., Manvell R.J., Brown I., Bestebroer T.M., van der Vliet S., Vuong O., Scheuer R.D., van der Jeugd H.P., Nolet B.A. (2016). Lack of virological and serological evidence for continued circulation of highly pathogenic avian influenza H5N8 virus in wild birds in the Netherlands, 14 November 2014 to 31 January 2016. Euro Surveill..

[154] Marchenko V.Y., Susloparov I.M., Kolosova N.P., Goncharova N.I., Shipovalov A.V., Durymanov A.G., Ilyicheva T.N., Budatsirenova L.V., Ivanova V.K., Ignatyev G.A. (2015). Influenza A(H5N8) virus isolation in Russia, 2014. Arch. Virol..

[155] van den Brand J.M.A., Verhagen J.H., Veldhuis Kroeze E.J.B., van de Bildt M.W.G., Bodewes R., Herfst S., Richard M., Lexmond P., Bestebroer T.M., Fouchier R.A.M. (2018). Wild ducks excrete highly pathogenic avian influenza virus H5N8 (2014–2015) without clinical or pathological evidence of disease. Emerg. Microbes Infect..

[156] Harder T., Maurer-Stroh S., Pohlmann A., Starick E., Höreth-Böntgen D., Albrecht K., Pannwitz G., Teifke J., Gunalan V., Lee R.T. (2015). Influenza A(H5N8) Virus Similar to Strain in Korea Causing Highly Pathogenic Avian Influenza in Germany. Emerg. Infect. Dis..

[157] Germundsson A., Madslien K.I., Hjortaas M.J., Handeland K., Jonassen C.M. (2010). Prevalence and subtypes of influenza A viruses in wild waterfowl in Norway 2006–2007. Acta Vet. Scand..

[158] Hall J.S., Russell R.E., Franson J.C., Soos C., Dusek R.J., Allen R.B., Nashold S.W., TeSlaa J.L., Jónsson J.E., Ballard J.R. (2015). Avian Influenza Ecology in North Atlantic Sea Ducks: Not All Ducks Are Created Equal. PLoS ONE.

[159] Alexander D.J. (2007). Summary of avian influenza activity in Europe, Asia, Africa, and Australasia, 2002–2006. Avian Dis..

[160] Fouchier R.A., Olsen B., Bestebroer T.M., Herfst S., van der Kemp L., Rimmelzwaan G.F., Osterhaus A.D. (2003). Influenza A virus surveillance in wild birds in Northern Europe in 1999 and 2000. Avian Dis..

[161] Krauss S., Walker D., Pryor S.P., Niles L., Chenghong L., Hinshaw V.S., Webster R.G. (2004). Influenza A viruses of migrating wild aquatic birds in North America. Vector Borne Zoonotic Dis..

[162] Krauss S., Obert C.A., Franks J., Walker D., Jones K., Seiler P., Niles L., Pryor S.P., Obenauer J.C., Naeve C.W. (2007). Influenza in migratory birds and evidence of limited intercontinental virus exchange. PLoS Pathog..

[163] Wilson H.M., Hall J.S., Flint P.L., Franson J.C., Ely C.R., Schmutz J.A., Samuel M.D. (2013). High seroprevalence of antibodies to avian influenza viruses among wild waterfowl in Alaska: Implications for surveillance. PLoS ONE.

[164] Pohlmann A., Starick E., Harder T., Grund C., Höper D., Globig A., Staubach C., Dietze K., Strebelow G., Ulrich R.G. (2017). Outbreaks among Wild Birds and Domestic Poultry Caused by Reassorted Influenza A(H5N8) Clade 2.3.4.4 Viruses, Germany, 2016. Emerg. Infect. Dis..

[165] Keawcharoen J., van Riel D., van Amerongen G., Bestebroer T., Beyer W.E., van Lavieren R., Osterhaus A.D., Fouchier R.A., Kuiken T. (2008). Wild ducks as long-distance vectors of highly pathogenic avian influenza virus (H5N1). Emerg. Infect. Dis..

[166] Spackman E., Prosser D.J., Pantin-Jackwood M.J., Berlin A.M., Stephens C.B. (2017). The Pathogenesis of Clade 2.3.4.4 H5 Highly Pathogenic Avian Influenza Viruses in Ruddy Duck (*Oxyura jamaicensis*) And Lesser Scaup (*Aythya affinis*). J. Wildl. Dis..

[167] CMS FAO CO-Convened Scientific Task Force on Avian Influenza and Wild Birds (2022). Scientific Task. Force on Avian Influenza and Wild Birds Statement. H5N1 Highly Pathogenic Avian Influenza in Poultry and Wild Birds: Winter of 2021/2022 with Focus on Mass Mortality of Wild Birds in UK and Israel.

[168] Food and Agriculture Organization of the United Nations Animal Health Global Avian Influenza Viruses with Zoonotic Potential. Situation Update. https://www.fao.org/animal-health/situation-updates/global-aiv-with-zoonotic-potential/en.

[169] Waldenström J., van Toor M., Lewis N., Lopes S., Javakhishvili Z., Muzika D., Fouchier R.A.M., Brouwer A. (2022). Active wild bird surveillance of avian influenza viruses, a report. EFSA Support. Publ..

[170] Floyd T., Banyard A.C., Lean F.Z.X., Byrne A.M.P., Fullick E., Whittard E., Mollett B.C., Bexton S., Swinson V., Macrelli M. (2021). Encephalitis and Death in Wild Mammals at a Rehabilitation Center after Infection with Highly Pathogenic Avian Influenza A(H5N8) Virus, United Kingdom. Emerg. Infect. Dis..

[171] Globig A., Staubach C., Sauter-Louis C., Dietze K., Homeier-Bachmann T., Probst C., Gethmann J., Depner K.R., Grund C., Harder T.C. (2017). Highly Pathogenic Avian Influenza H5N8 Clade 2.3.4.4b in Germany in 2016/2017. Front. Vet. Sci..

[172] Peyrot B.M., Abolnik C., Anthony T., Roberts L.C. (2022). Evolutionary dynamics of the clade 2.3.4.4B H5N8 high-pathogenicity avian influenza outbreaks in coastal seabirds and other species in southern Africa from 2017 to 2019. Transbound. Emerg. Dis..

[173] McPhail G.M., Collins S.M., Burt T.V., Careen N.G., Doiron P.B., Avery-Gomm S., Barychka T., English M.D., Giacinti J.A., Jones M.E.B. (2024). Geographic, ecological, and temporal patterns of seabird mortality during the 2022 HPAI H5N1 outbreak on the island of Newfoundland. bioRxiv.

[174] Rijks J.M., Leopold M.F., Kühn S., In ‘t Veld R., Schenk F., Brenninkmeijer A., Lilipaly S.J., Ballmann M.Z., Kelder L., de Jong J.W. (2022). Mass Mortality Caused by Highly Pathogenic Influenza A(H5N1) Virus in Sandwich Terns, The Netherlands, 2022. Emerg. Infect. Dis..

[175] Abolnik C., Pieterse R., Peyrot B.M., Choma P., Phiri T.P., Ebersohn K., Heerden C.J.V., Vorster A.A., Zel G.V., Geertsma P.J. (2019). The Incursion and Spread of Highly Pathogenic Avian Influenza H5N8 Clade 2.3.4.4 Within South Africa. Avian Dis..

[176] Banyard A.C., Lean F.Z.X., Robinson C., Howie F., Tyler G., Nisbet C., Seekings J., Meyer S., Whittard E., Ashpitel H.F. (2022). Detection of Highly Pathogenic Avian Influenza Virus H5N1 Clade 2.3.4.4b in Great Skuas: A Species of Conservation Concern in Great Britain. Viruses.

[177] Camphuysen C., Gear S., Furness B. (2022). Avian influenza leads to mass mortality of adult Great Skuas in Foula in summer 2022. Scott Birds.

[178] Olsen B., Munster V.J., Wallensten A., Waldenström J., Osterhaus A.D., Fouchier R.A. (2006). Global patterns of influenza a virus in wild birds. Science.

[179] Wille M., Lisovski S., Risely A., Ferenczi M., Roshier D., Wong F.Y.K., Breed A.C., Klaassen M., Hurt A.C. (2019). Serologic Evidence of Exposure to Highly Pathogenic Avian Influenza H5 Viruses in Migratory Shorebirds, Australia. Emerg. Infect. Dis..

[180] Fusaro A., Zecchin B., Vrancken B., Abolnik C., Ademun R., Alassane A., Arafa A., Awuni J.A., Couacy-Hymann E., Coulibaly M.B. (2019). Disentangling the role of Africa in the global spread of H5 highly pathogenic avian influenza. Nat. Comm..

[181] Molini U., Aikukutu G., Roux J.P., Kemper J., Ntahonshikira C., Marruchella G., Khaiseb S., Cattoli G., Dundon W.G. (2020). Avian Influenza H5N8 Outbreak in African Penguins (*Spheniscus demersus*), Namibia, 2019. J. Wildl. Dis..

[182] Molini U., Yabe J., Meki I.K., Ouled Ahmed Ben Ali H., Settypalli T.B.K., Datta S., Coetzee L.M., Hamunyela E., Khaiseb S., Cattoli G. (2023). Highly pathogenic avian influenza H5N1 virus outbreak among Cape cormorants (*Phalacrocorax capensis*) in Namibia, 2022. Emerg. Microbes Infect..

[183] Beyit A.D., Meki I.K., Barry Y., Haki M.L., El Ghassem A., Hamma S.M., Abdelwahab N., Doumbia B., Ahmed Benane H., Daf D.S. (2023). Avian influenza H5N1 in a great white pelican (*Pelecanus onocrotalus*), Mauritania 2022. Vet. Res. Commun..

[184] Bertran K., Dolz R., Majó N. (2014). Pathobiology of avian influenza virus infection in minor gallinaceous species: A review. Avian Pathol..

[185] Grant M., Bröjer C., Zohari S., Nöremark M., Uhlhorn H., Jansson D.S. (2022). Highly Pathogenic Avian Influenza (HPAI H5Nx, Clade 2.3.4.4.b) in Poultry and Wild Birds in Sweden: Synopsis of the 2020–2021 Season. Vet. Sci..

[186] Tammiranta N., Isomursu M., Fusaro A., Nylund M., Nokireki T., Giussani E., Zecchin B., Terregino C., Gadd T. (2023). Highly pathogenic avian influenza A (H5N1) virus infections in wild carnivores connected to mass mortalities of pheasants in Finland. Infect. Genet. Evol..

[187] Liang Y., Hjulsager C.K., Seekings A.H., Warren C.J., Lean F.Z.X., Núñez A., James J., Thomas S.S., Banyard A.C., Slomka M.J. (2022). Pathogenesis and infection dynamics of high pathogenicity avian influenza virus (HPAIV) H5N6 (clade 2.3.4.4b) in pheasants and onward transmission to chickens. Virology.

[188] Brookes S.M., Mansfield K.L., Reid S.M., Coward V., Warren C., Seekings J., Brough T., Gray D., Núñez A., Brown I.H. (2022). Incursion of H5N8 high pathogenicity avian influenza virus (HPAIV) into gamebirds in England. Epidemiol. Infect..

[189] Stoimenov G.M., Goujgoulova G.V., Nikolov B., Hristov K., Teneva A. (2019). Pathological Changes in Natural Infection of Pheasants with Highly Pathogenic Avian Influenza A (H5N8) in Bulgaria. J. Vet. Res..

[190] Abolnik C. (2014). A current review of avian influenza in pigeons and doves (*Columbidae*). Vet. Microbiol..

[191] Jeong S., Kwon J.H., Lee S.H., Kim Y.J., Jeong J.H., Park J.E., Jheong W.H., Lee D.H., Song C.S. (2021). Subclinical Infection and Transmission of Clade 2.3.4.4 H5N6 Highly Pathogenic Avian Influenza Virus in Mandarin Duck (*Aix galericulata*) and Domestic Pigeon (*Columbia livia domestica*). Viruses.

[192] Peters M., King J., Wohlsein P., Grund C., Harder T. (2022). Genuine lethal infection of a wood pigeon (*Columba palumbus*) with high pathogenicity avian influenza H5N1, clade 2.3.4.4b, in Germany, 2022. Vet. Microbiol..

[193] Shriner S.A., Root J.J. (2020). A Review of Avian Influenza A Virus Associations in Synanthropic Birds. Viruses.

[194] Krone O., Globig A., Ulrich R., Harder T., Schinköthe J., Herrmann C., Gerst S., Conraths F.J., Beer M. (2018). White-Tailed Sea Eagle (*Haliaeetus albicilla*) Die-Off Due to Infection with Highly Pathogenic Avian Influenza Virus, Subtype H5N8, in Germany. Viruses.

[195] Günther A., Pohlmann A., Globig A., Ziegler U., Calvelage S., Keller M., Fischer D., Staubach C., Groschup M.H., Harder T. (2023). Continuous surveillance of potentially zoonotic avian pathogens detects contemporaneous occurrence of highly pathogenic avian influenza viruses (HPAIV H5) and flaviviruses (USUV, WNV) in several wild and captive birds. Emerg. Microbes Infect..

[196] Letsholo S.L., James J., Meyer S.M., Byrne A.M.P., Reid S.M., Settypalli T.B.K., Datta S., Oarabile L., Kemolatlhe O., Pebe K.T. (2022). Emergence of High Pathogenicity Avian Influenza Virus H5N1 Clade 2.3.4.4b in Wild Birds and Poultry in Botswana. Viruses.

[197] Caliendo V., Leijten L., van de Bildt M.W.G., Fouchier R.A.M., Rijks J.M., Kuiken T. (2022). Pathology and virology of natural highly pathogenic avian influenza H5N8 infection in wild Common buzzards (*Buteo buteo*). Sci. Rep..

[198] Swayne D.E., Suarez D.L., Sims L.D. (2020). Influenza. Diseases of Poultry.

[199] Penski N., Härtle S., Rubbenstroth D., Krohmann C., Ruggli N., Schusser B., Pfann M., Reuter A., Gohrbandt S., Hundt J. (2011). Highly pathogenic avian influenza viruses do not inhibit interferon synthesis in infected chickens but can override the interferon-induced antiviral state. J. Virol..

[200] Cao Y., Huang Y., Xu K., Liu Y., Li X., Xu Y., Zhong W., Hao P. (2017). Differential responses of innate immunity triggered by different subtypes of influenza a viruses in human and avian hosts. BMC Med. Genom..

[201] Moulin H.R., Liniger M., Python S., Guzylack-Piriou L., Ocaña-Macchi M., Ruggli N., Summerfield A. (2011). High interferon type I responses in the lung, plasma and spleen during highly pathogenic H5N1 infection of chicken. Vet. Res..

[202] Park M.-J., Cha R.M., Kye S.-J., Lee Y.-N., Kim N.-Y., Baek Y.-G., Heo G.-B., Sagong M., Lee K.-N., Lee Y.-J. (2021). Pathogenicity of H5N8 High Pathogenicity Avian Influenza Virus in Chickens and Ducks from South Korea in 2020–2021. Viruses.

[203] Sánchez-González R., Ramis A., Nofrarías M., Wali N., Valle R., Pérez M., Perlas A., Majó N. (2020). Pathobiology of the highly pathogenic avian influenza viruses H7N1 and H5N8 in different chicken breeds and role of Mx 2032 G/A polymorphism in infection outcome. Vet. Res..

[204] Yehia N., Erfan A.M., Adel A., El-Tayeb A., Hassan W.M.M., Samy A., Abd El-Hack M.E., El-Saadony M.T., El-Tarabily K.A., Ahmed K.A. (2022). Pathogenicity of three genetically distinct and highly pathogenic Egyptian H5N8 avian influenza viruses in chickens. Poult. Sci..

[205] Pantin-Jackwood M.J., Spackman E., Leyson C., Youk S., Lee S.A., Moon L.M., Torchetti M.K., Killian M.L., Lenoch J.B., Kapczynski D.R. (2023). Pathogenicity in Chickens and Turkeys of a 2021 United States H5N1 Highly Pathogenic Avian Influenza Clade 2.3.4.4b Wild Bird Virus Compared to Two Previous H5N8 Clade 2.3.4.4 Viruses. Viruses.

[206] Prokopyeva E.A., Zinserling V.A., Bae Y.C., Kwon Y., Kurskaya O.G., Sobolev I.A., Kozhin P.M., Komissarov A., Fadeev A., Petrov V. (2019). Pathology of A(H5N8) (Clade 2.3.4.4) Virus in Experimentally Infected Chickens and Mice. Interdiscip. Perspect. Infect. Dis..

[207] Matsuu A., Tanikawa T., Fujimoto Y., Yabuki M., Tsunekuni R., Sakuma S., Uchida Y., Saito T. (2021). Different Sensitivity of Japanese Native-Bred Chickens to H5 Subtypes of Highly Pathogenic Avian Influenza Viruses. Avian Dis..

[208] Blohm U., Weigend S., Preisinger R., Beer M., Hoffmann D. (2016). Immunological Competence of Different Domestic Chicken Breeds Against Avian Influenza Infection. Avian Dis..

[209] Spickler A.R., Trampel D.W., Roth J.A. (2008). The onset of virus shedding and clinical signs in chickens infected with high-pathogenicity and low-pathogenicity avian influenza viruses. Avian Pathol..

[210] Mosad S.M., El-Gohary F.A., Ali H.S., El-Sharkawy H., Elmahallawy E.K. (2020). Pathological and Molecular Characterization of H5 Avian Influenza Virus in Poultry Flocks from Egypt over a Ten-Year Period (2009–2019). Animals.

[211] Bertran K., Swayne D.E., Pantin-Jackwood M.J., Kapczynski D.R., Spackman E., Suarez D.L. (2016). Lack of chicken adaptation of newly emergent Eurasian H5N8 and reassortant H5N2 high pathogenicity avian influenza viruses in the U.S. is consistent with restricted poultry outbreaks in the Pacific flyway during 2014–2015. Virology.

[212] DeJesus E., Costa-Hurtado M., Smith D., Lee D.H., Spackman E., Kapczynski D.R., Torchetti M.K., Killian M.L., Suarez D.L., Swayne D.E. (2016). Changes in adaptation of H5N2 highly pathogenic avian influenza H5 clade 2.3.4.4 viruses in chickens and mallards. Virology.

[213] Sironi L., Williams J.L., Moreno-Martin A.M., Ramelli P., Stella A., Jianlin H., Weigend S., Lombardi G., Cordioli P., Mariani P. (2008). Susceptibility of different chicken lines to H7N1 highly pathogenic avian influenza virus and the role of Mx gene polymorphism coding amino acid position 631. Virology.

[214] Matsui M., Moriya O., Belladonna M.L., Kamiya S., Lemonnier F.A., Yoshimoto T., Akatsuka T. (2004). Adjuvant activities of novel cytokines, interleukin-23 (IL-23) and IL-27, for induction of hepatitis C virus-specific cytotoxic T lymphocytes in HLA-A*0201 transgenic mice. J. Virol..

[215] Park S.C., Song B.M., Lee Y.N., Lee E.K., Heo G.B., Kye S.J., Lee K.H., Bae Y.C., Lee Y.J., Kim B. (2019). Pathogenicity of clade 2.3.4.4 H5N6 highly pathogenic avian influenza virus in three chicken breeds from South Korea in 2016/2017. J. Vet. Sci..

[216] Suba S., Nagarajan S., Saxena V.K., Kumar M., Vanamayya P.R., Rajukumar K., Gowthaman V., Jain V., Singh D.P., Dubey S.C. (2015). Pathology of a H5N1, highly pathogenic avian influenza virus, in two Indian native chicken breeds and a synthetic broiler line. Indian J. Exp. Biol..

[217] Tumpey T.M., Kapczynski D.R., Swayne D.E. (2004). Comparative susceptibility of chickens and turkeys to avian influenza A H7N2 virus infection and protective efficacy of a commercial avian influenza H7N2 virus vaccine. Avian Dis..

[218] Aldous E.W., Seekings J.M., McNally A., Nili H., Fuller C.M., Irvine R.M., Alexander D.J., Brown I.H. (2010). Infection dynamics of highly pathogenic avian influenza and virulent avian paramyxovirus type 1 viruses in chickens, turkeys and ducks. Avian Pathol..

[219] Malmberg J.L., Miller M., Jennings-Gaines J., Allen S.E. (2023). Mortality in Wild Turkey (Meleagris gallopavo) Associated with Natural Infection with H5N1 Highly Pathogenic Avian Influenza Virus (HPAIV) Subclade 2.3.4.4. J. Wildl. Dis..

[220] Anis A., AboElkhair M., Ibrahim M. (2018). Characterization of highly pathogenic avian influenza H5N8 virus from Egyptian domestic waterfowl in 2017. Avian Pathol..

[221] Sun H., Pu J., Hu J., Liu L., Xu G., Gao G.F., Liu X., Liu J. (2016). Characterization of clade 2.3.4.4 highly pathogenic H5 avian influenza viruses in ducks and chickens. Vet. Microbiol..

[222] Bányai K., Bistyák A.T., Thuma Á., Gyuris É., Ursu K., Marton S., Farkas S.L., Hortobágyi E., Bacsadi Á., Dán Á. (2016). Neuroinvasive influenza virus A(H5N8) in fattening ducks, Hungary, 2015. Infect. Genet. Evol..

[223] Foret-Lucas C., Figueroa T., Coggon A., Houffschmitt A., Dupré G., Fusade-Boyer M., Guérin J.-L., Delverdier M., Bessière P., Volmer R. (2023). In Vitro and In Vivo Characterization of H5N8 High-Pathogenicity Avian Influenza Virus Neurotropism in Ducks and Chickens. Microbiol. Spectr..

[224] Beerens N., Heutink R., Bergervoet S.A., Harders F., Bossers A., Koch G. (2017). Multiple Reassorted Viruses as Cause of Highly Pathogenic Avian Influenza A(H5N8) Virus Epidemic, the Netherlands, 2016. Emerg. Infect. Dis..

[225] Scheibner D., Breithaupt A., Luttermann C., Blaurock C., Mettenleiter T.C., Abdelwhab E.M. (2022). Genetic Determinants for Virulence and Transmission of the Panzootic Avian Influenza Virus H5N8 Clade 2.3.4.4 in Pekin Ducks. J. Virol..

[226] Zhu Y., Cong Y., Sun Y., Han J., Gai L., Yang T., Liu C., Zhao L., Cong Y. (2023). Isolation and Identification of Novel Highly Pathogenic Avian Influenza Virus (H5N8) Subclade 2.3.4.4b from Geese in Northeastern China. Appl. Environ. Microbiol..

[227] Animal and Plant Health Inspection Service, U.S. Department of Agriculture 2022–2023 Detections of Highly Pathogenic Avian Influenza. https://www.aphis.usda.gov/livestock-poultry-disease/avian/avian-influenza/hpai-detections.

[228] Agüero M., Monne I., Sánchez A., Zecchin B., Fusaro A., Ruano M.J., del Valle Arrojo M., Fernández-Antonio R., Souto A.M., Tordable P. (2023). Highly pathogenic avian influenza A(H5N1) virus infection in farmed minks, Spain, October 2022. Euro Surveill..

[229] Lindh E., Lounela H., Ikonen N., Kantala T., Savolainen-Kopra C., Kauppinen A., Österlund P., Kareinen L., Katz A., Nokireki T. (2023). Highly pathogenic avian influenza A(H5N1) virus infection on multiple fur farms in the South and Central Ostrobothnia regions of Finland, July 2023. Euro Surveill..

[230] Hervé S., Schmitz A., Briand F.X., Gorin S., Quéguiner S., Niqueux É., Paboeuf F., Scoizec A., Le Bouquin-Leneveu S., Eterradossi N. (2021). Serological Evidence of Backyard Pig Exposure to Highly Pathogenic Avian Influenza H5N8 Virus during 2016-2017 Epizootic in France. Pathogens.

[231] Rosone F., Bonfante F., Sala M.G., Maniero S., Cersini A., Ricci I., Garofalo L., Caciolo D., Denisi A., Napolitan A. (2023). Seroconversion of a Swine Herd in a Free-Range Rural Multi-Species Farm against HPAI H5N1 2.3.4.4b Clade Virus. Microorganisms.

[232] Domańska-Blicharz K., Świętoń E., Świątalska A., Monne I., Fusaro A., Tarasiuk K., Wyrostek K., Styś-Fijoł N., Giza A., Pietruk M. (2023). Outbreak of highly pathogenic avian influenza A(H5N1) clade 2.3.4.4b virus in cats, Poland, June to July 2023. Euro Surveill..

[233] Centers for Disease Control and Prevention Ask the Expert: Highly Pathogenic Avian Influenza A(H5N1) Viruses. https://www.cdc.gov/flu/avianflu/spotlights/2022-2023/avian-flu-highly-pathogenic.htm.

[234] Centers for Disease Control and Prevention, National Center for Immunization and Respiratory Diseases (NCIRD) Human Infection with Highly Pathogenic Avian Influenza A(H5N1) Virus in Chile. https://www.cdc.gov/flu/avianflu/spotlights/2022-2023/chile-first-case-h5n1-addendum.htm.

[235] Gabriel G., Czudai-Matwich V., Klenk H.D. (2013). Adaptive mutations in the H5N1 polymerase complex. Virus Res..

[236] Ma W., Kahn R.E., Richt J.A. (2008). The pig as a mixing vessel for influenza viruses: Human and veterinary implications. J. Mol. Genet. Med..

[237] Graaf A., Piesche R., Sehl-Ewert J., Grund C., Pohlmann A., Beer M., Harder T. (2023). Low Susceptibility of Pigs against Experimental Infection with HPAI Virus H5N1 Clade 2.3.4.4b. Emerg. Infect. Dis..

[238] Centers for Disease Control and Prevention, National Center for Immunization and Respiratory Diseases (NCIRD) Current H5N1 Bird Flu Situation in Dairy Cows. https://www.cdc.gov/flu/avianflu/mammals.htm#:~:text=CDC%20confirmed%20one%20human%20HPAI,likely%20mammal%20to%20human%20transmission.

[239] American Veterinary Medical Association (AVMA) Highly Pathogenic Avian Influenza Detected in TX, KS Dairy Cattle. https://www.avma.org/news/press-releases/highly-pathogenic-avian-influenza-detected-tx-ks-dairy-cattle.

[240] Centers for Disease Control and Prevention Highly Pathogenic Avian Influenza A (H5N1) Virus Infection Reported in a Person in the U.S. https://www.cdc.gov/media/releases/2024/p0401-avian-flu.html.

[241] American Veterinary Medical Association (AVMA) Goat in Minnesota Tests Positive for HPAI. https://www.avma.org/news/goat-minnesota-tests-positive-hpai#:~:text=On%20March%2020%2C%20the%20Minnesota,virus%20in%20a%20domestic%20ruminant.

[242] Centers for Disease Control and Prevention, National Center for Immunization and Respiratory Diseases (NCIRD) Considerations for Veterinarians: Evaluating and Handling of Cats Potentially Exposed to Highly Pathogenic Avian Influenza A(H5N1) Virus. https://www.cdc.gov/flu/avianflu/veterinarians-handling-cats.htm.

[243] Briand F.X., Souchaud F., Pierre I., Beven V., Hirchaud E., Hérault F., Planel R., Rigaudeau A., Bernard-Stoecklin S., Van der Werf S. (2023). Highly Pathogenic Avian Influenza A(H5N1) Clade 2.3.4.4b Virus in Domestic Cat, France, 2022. Emerg. Infect. Dis..

[244] Shin D.L., Siebert U., Lakemeyer J., Grilo M., Pawliczka I., Wu N.H., Valentin-Weigand P., Haas L., Herrler G. (2019). Highly Pathogenic Avian Influenza A(H5N8) Virus in Gray Seals, Baltic Sea. Emerg. Infect. Dis..

[245] Postel A., King J., Kaiser F.K., Kennedy J., Lombardo M.S., Reineking W., de le Roi M., Harder T., Pohlmann A., Gerlach T. (2022). Infections with highly pathogenic avian influenza A virus (HPAIV) H5N8 in harbor seals at the German North Sea coast, 2021. Emerg. Microbes Infect..

[246] Puryear W., Sawatzki K., Hill N., Foss A., Stone J.J., Doughty L., Walk D., Gilbert K., Murray M., Cox E. (2023). Highly Pathogenic Avian Influenza A(H5N1) Virus Outbreak in New England Seals, United States. Emerg. Infect. Dis..

[247] Gamarra-Toledo V., Plaza P.I., Gutiérrez R., Luyo P., Hernani L., Angulo F., Lambertucci S.A. (2023). Avian flu threatens Neotropical birds. Science.

[248] Murawski A., Fabrizio T., Ossiboff R., Kackos C., Jeevan T., Jones J., Kandeil A., Walker D., Turner J., Patton C. (2023). Highly pathogenic avian influenza A(H5N1) virus in a common bottlenose dolphin (*Tursiops truncatus*) in Florida. Commun. Biol..

[249] Thorsson E., Zohari S., Roos A., Banihashem F., Bröjer C., Neimanis A. (2023). Highly Pathogenic Avian Influenza A(H5N1) Virus in a Harbor Porpoise, Sweden. Emerg. Infect. Dis..

[250] Yang Z.Y., Wei C.J., Kong W.P., Wu L., Xu L., Smith D.F., Nabel G.J. (2007). Immunization by avian H5 influenza hemagglutinin mutants with altered receptor binding specificity. Science.

[251] Animal and Plant Health Inspection Service, U.S. Department of Agriculture Detections of Highly Pathogenic Avian Influenza in Mammals. https://www.aphis.usda.gov/livestock-poultry-disease/avian/avian-influenza/hpai-detections/mammals.

[252] World Organization for Animal Health Cases of Avian Influenza in Mammals. https://www.woah.org/en/disease/avian-influenza/#ui-id-2.

[253] Bordes L., Vreman S., Heutink R., Roose M., Venema S., Pritz-Verschuren S.B.E., Rijks J.M., Gonzales J.L., Germeraad E.A., Engelsma M. (2023). Highly Pathogenic Avian Influenza H5N1 Virus Infections in Wild Red Foxes (*Vulpes vulpes*) Show Neurotropism and Adaptive Virus Mutations. Microbiol. Spectr..

[254] Reperant L.A., van Amerongen G., van de Bildt M.W., Rimmelzwaan G.F., Dobson A.P., Osterhaus A.D., Kuiken T. (2008). Highly pathogenic avian influenza virus (H5N1) infection in red foxes fed infected bird carcasses. Emerg. Infect. Dis..

[255] Hiono T., Kobayashi D., Kobayashi A., Suzuki T., Satake Y., Harada R., Matsuno K., Sashika M., Ban H., Kobayashi M. (2023). Virological, pathological, and glycovirological investigations of an Ezo red fox and a tanuki naturally infected with H5N1 high pathogenicity avian influenza viruses in Hokkaido, Japan. Virology.

